# Prefrontal modulation of striatal engagement: a hierarchical framework for goal-directed behavior

**DOI:** 10.1038/s42003-026-10047-9

**Published:** 2026-05-06

**Authors:** Aron Koszeghy, Johannes Passecker

**Affiliations:** Institute of Systems Neuroscience, Medical University of Innsbruck, Innsbruck, Austria

**Keywords:** Neural circuits, Cognitive control, Cortex, Reward

## Abstract

We review Prefrontal-striatum interplay in goal-directed behavior, focusing on prefrontal (PFC) and striatal macro- and micro-domain interactions in hierarchical engagement with goals, strategies and actions. This dual-control framework posits a role for the striatal matrix in engaging with behavioral options- for automatization, and prefrontal modulation of striatal engagement for flexibility. Hierarchical prefronto-basal-ganglia loops incorporating ventral-, dorsomedial-, and dorsolateral striatum represent goals, strategies and actions respectively. The striatal “switchboard” enables engagement, a unified function across macro-domains, while its modulation by PFC operates through dual mechanisms: fast switching via direct matrix inputs, and slower, learning-based adaptation using a striosome-dopamine pathway.

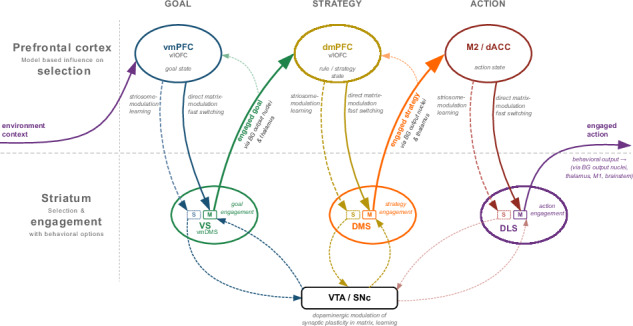

## Abstract (web summary)

Goal-directed behavior requires selecting and committing to goals, strategies, and actions. This review explores how three hierarchical prefronto-striatal loops can achieve this: striatal matrix ensembles maintain engagement, prefrontal inputs bias selection.

## Introduction and perspective of the conceptual review

The prefrontal cortex (PFC) and the striatum, the main input nucleus of the basal ganglia (BG), are crucial for various aspects of goal-directed behavior^1–3^. While these regions have been extensively studied in isolation, understanding how they collaborate to drive flexible decision-making remains a challenge. Historically, this gap was due to methodological limitations, but recent advancements such as multi silicon probe recordings, cell type-specific expression methods and optogenetics have begun to illuminate these complex interactions^1,4–6^. A comprehensive understanding requires considering the prefrontal and striatal circuits not as isolated units, but as parts of an integrated system that considers most, if not all, of the prefrontal and striatal subdomains at the same time.

This review focuses on prefrontal and striatal areas with shared homologs in rodents and primates (see Table 1 for a comprehensive list). In the rodent, the PFC includes the prelimbic (PrL, A32), anterior cingulate (ACC, A24), medial- ventral- and lateral- orbitofrontal (m/v/lOFC, MO A14, VO A13a, LO A13l/m), and infralimbic (IL, A25) cortices^7–9^. The striatum is functionally divided into three macrodomains: the dorsomedial (DMS), ventral (VS), and dorsolateral (DLS) striatum. Internally, these macrodomains are further compartmentalized into striosomes (patches) and the matrix. Another layer of striatal organization involves the distinction between direct pathway (D1R-MSN) and indirect pathway (D2R-MSN) medium spiny neurons (MSN). In coordinated opposition, the direct pathway facilitates the selected, fitting, action (behavioral option), while the indirect pathway suppresses competing alternatives; with their relative balance determining together the engaged behavioral option^10–12^. Both cell types are found within striosome and matrix compartments^13,14^, in differing proportions and reviewed elsewhere^15–21^. While we focus on the striatum, the largest input nucleus of BG, we briefly need to acknowledge the importance of other nuclei of BG. The Subthalamic Nucleus (STN), as the stand-alone input nucleus of the hyper direct pathway, has multiple suggested functions (which can supplement behavioral option engagement); including fast global inhibition of engaged behavioral options allowing post-hoc re-engagement with others^22,23^. The output nuclei (globus pallidus internus—GPi, Substantia Nigra pars reticulata—SNr) of BG, provide constant inhibition to their thalamic and brainstem targets, keeping the whole repertoire of behavioral options inactive (except the ones matching current situation). As cortical areas and the behavioral repertoire of different species change, the structure and relative importance of BG nuclei and sub-nuclei also change^24^, including species-specific differences in the topographic organization of GPi and SNr projections to thalamic nuclei involved in PFC-BG loops^25,26^.

**Table 1:**
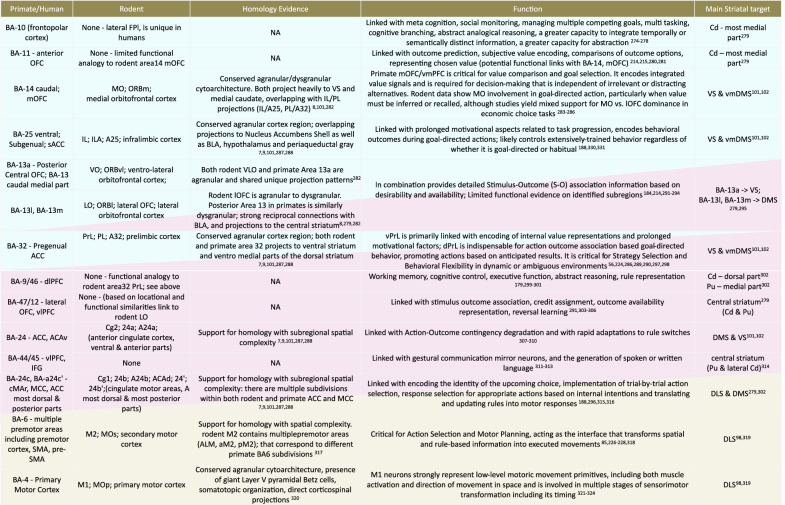
Function and Striatal Projections of Analogous Prefrontal Areas in Rodents and Primates

While this review focuses on prefrontal and striatal regions conserved across species, this table also includes primate-specific PFC regions for context. It is important to note that while anatomical atlases traditionally often define sharp boundaries useful for simplified models, biological organization in both the PFC and striatum often follows functional and connectivity-based gradients that span these borders^99,188,190^. Here, Brodmann Areas are organized into three broad functional domains. Rodent terminology incorporates nomenclature from the Paxinos, Swanson, and Allen atlases. Color hues denote Goal(blue), Strategy (pink) and Action (gray) selection and engagement related larger anatomical units, which in rodents map on to vmPFC (& v/lOFC), dmPFC (& v/lOFC), and motor cortices, respectively.

*VS* Ventral Striatum, *DMS* Dorsomedial Striatum, *DLS* Dorsolateral Striatum; *S-O* Stimulus-Outcome, *A-O* Action-Outcome, *OFC* Orbitofrontal Cortex, *ACC* Anterior Cingulate Cortex; *MCC* Midcingulate Cortex, *BLA* Basolateral Amygdala; *NAc* Nucleus Accumbens, *PAG* Periaqueductal Gray, *vmPFC* ventromedial Prefrontal Cortex, *dlPFC* dorsolateral Prefrontal Cortex, *vlPFC* ventrolateral Prefrontal Cortex, *IFG* Inferior Frontal Gyrus, *SMA* Supplementary Motor Area, *cMAr* rostral cingulate motor area, *vmDMS* Dorsomedial Striatum (most ventral and medial aspects), *Cd* Caudate nucleus, *Pu* Putamen.

Our analysis centers on goal-directed behavior (often used interchangeably with “model-based” behavior), which is characterized by its deliberate and informed nature. It is distinguished from habitual behaviors by key features, including sensitivity to current outcome value^27^ and the capacity to generate novel action sequences to achieve a desired outcome^28^. Crucially, it relies on an “internal world model” representing the causal structure of the environment, allowing agents to predict consequences and guide their actions^3,29–31^.

To examine the interplay between the PFC and striatum in supporting goal-directed behavior, we propose an integrative framework built on five core concepts: i) Hierarchical Selection: Goals, strategies, and actions are selected and engaged concurrently via serially connected PFC-BG loops. ii) Unified Matrix Function: Across all macrodomains, striatal matrix ensembles function to maintain engagement (temporal commitment) with a selected behavioral option. This mechanism allows for the automatization of specific behaviors, whether goals, strategies, or actions, without requiring continuous attention during stable periods. iii) Associative Learning: Learning is enabled across all striatal macrodomains by two key features: the high convergence of synaptic inputs from various cortical and subcortical (including thalamic) areas onto matrix MSNs, and the capacity for dopamine-dependent plasticity at these synapses. iv) Evaluative Learning Loop: The PFC influences learning by providing evaluative input to striosomes. In turn, striosomes regulate midbrain dopamine neurons, which modulate plasticity within the matrix. v) Real-time Switching: Direct PFC synaptic inputs to the matrix exert real-time control over behavior. These inputs can either initiate engagement by activating a specific learned association (an MSN ensemble) or rapidly switch activity to a different ensemble, thereby determining the currently selected and engaged behavioral option.

**Fig. 1:**
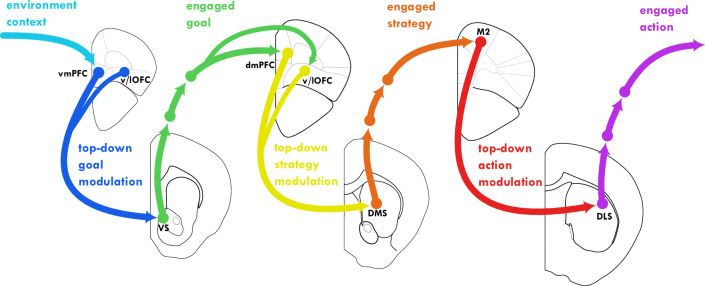
Hierarchical Selection and Engagement with Goals, Strategies, and Actions via Prefrontal cortex Basal Ganglia (CBG) Loops. This figure illustrates (from left to right) how hierarchical engagement with goals, strategies, and actions is achieved through serially connected CBG loops. Each striatal macrodomain region receives topographically organized input from specific cortical areas. Through the output nuclei of the BG and thalamus, these striatal regions project non-exclusively back to their respective cortical input areas forming CBG loops. The sequential flow of information through these interconnected loops supports hierarchical striatal engagement with multiple behavioral options. Note that the two rows represent the prefronto-striatal dual-control over behavior, upward arrows pointing away from the striatum represent behavioral-engagement, downward arrows pointing away from PFC represent top-down modulation of engagement. To emphasize the information flow between loops, the recurrent connections returning to the originating cortical regions are not shown. Brain illustrations are based on The Allen Mouse Brain CCF^271,272,273^. vmPFC stands for ventro-medial prefrontal cortex, v/lOFC stands for ventral and lateral orbitofrontal cortex (see also Table 1 and main text).

By integrating these concepts, we outline a theoretical model (Fig. 1) in which the PFC and striatum cooperate continuously to balance stable engagement with flexible adaptation. We critically evaluate the evidence supporting this integrated model and highlight key areas for future research.

## A dual-control architecture for automatization and flexibility

What is the role of prefrontal-striatal communication in goal-directed behavior? We propose that it facilitates optimized engagement with behavioral options. We derive this terminology from Hierarchical Reinforcement Learning^32^ as a closed-loop control policy that extends over time. In this framework, selecting a behavioral option implies engaging with it, maintaining its execution until a specific termination condition is met. This applies hierarchically to actions, strategies, and goals. This is in line with, and extending beyond the motoric domain, of the basal ganglia (BG), and within that striatum (its primary input nucleus), as a programmable ‘switchboard’ that represents the entire action space^33–35^. The striatum’s primary role is not just to initiate actions, but to select and maintain engagement with specific behavioral options, thereby regulating their onset, duration and offset^34,36–39^. This selection occurs predominantly via cortico-striatal and thalamo-striatal synaptic input patterns activating striatal MSN ensembles, which in turn represent engagement through disinhibiting a chosen behavioral option from a pool of competing, inhibited alternatives^40–45^.

The PFC modulates this striatal ‘switchboard’ to initiate behavioral adaptation when environmental state changes necessitate it. This is achieved through two primary, dissociable pathways^1,5,30,46,47^.

1) Slower Learning Pathway: PFC projections to striosomes provide evaluative signals^46^. These striosomal projections, via their connections with midbrain dopaminergic neurons^48^, can indirectly modulate cortico-striatal and thalamo-striatal synaptic plasticity in the matrix^49–51^ (see also later sections), enabling striatum to learn, re-learn and adapt via re-routing associations between stimuli, actions, and outcomes^52–54^. We define this process as slow because it relies on dopaminergic synaptic plasticity (reinforcement learning) that is induced and expressed over minutes to hours, necessary for consolidating new associations.

2) Faster Switching Pathway: Direct PFC projections to the striatal matrix MSNs^14^ or interneurons^55^, convey information about the current state of the internal world model, enabling rapid, model-based switching between engaged behavioral options^3,30,31,56,57^. This is a faster process as it operates on the timescale of synaptic transmission and population dynamics (milliseconds to seconds). This allows for real-time flexibility, such as switching between pre-established strategies on a trial-by-trial basis, without the immediate need for synaptic plasticity. A potential supplementary mechanism for rapid behavioral inhibition is the cortico-subthalamic hyperdirect pathway, which can operate in parallel to these striatal circuits^58,59^.

This dual prefronto-striatal behavioral control architecture allows for both computationally efficient, automatized behavior when adjustments are not required, and enhanced behavioral flexibility when they are.

This automatization is expected to not be limited to the well established stimulus-response (S-R) habits of the DLS^60–65^; wherein, once associations are learnt, specific patterns of cortico-striatal and thalamo-striatal inputs can elicit motor actions by activating corresponding DLS MSN ensembles^66–72^, bypassing the need for conscious deliberation. Rather, we predict that the underlying neurobiological mechanism, associating specific input patterns with specific MSN ensemble activity, extends across all striatal macrodomains and thus likely to other domains of the extended behavioral-option space^73^. Consequently, engagement is a unified function that applies hierarchically to goals, strategies, and actions (Fig. 1), which are implemented via interconnected CBG loops^74–80^. Anatomical evidence shows these loops are not only recurrent via the thalamus but also connect to other cortical areas^78,81–83^, creating the substrate for hierarchical sequential selection of multiple behavioral options, and maintaining concurrent engagement with them. Such hierarchy consists of three layers: i) Goal Engagement: A ventromedial PFC (vmPFC)-VS loop selects and maintains engagement with a goal based on the current context^84^. Here, the term “goal” refers to a desired future state that an organism is actively pursuing, dependent on an anticipated outcome state itself (not for example a distinct spatial location delivering the outcome). ii) Strategy Engagement: A dorsomedial PFC (dmPFC)/lateral OFC-DMS loop then selects and engages with the optimal strategy to achieve that goal^1,4^. Strategy is defined here as an association between a desired outcome and outcome-related context with either an outcome-predictive stimulus, or an outcome-predictive response-type. These associations guide behavior by specifying which response-type or sensory stimulus to select given an engaged outcome, and specific environmental/task contexts, to achieve the desired outcome. iii) Action Engagement: Finally, a motor cortices/dorsal ACC-DLS loop selects and engages with the action that implements the chosen strategy^85^. Actions are simple motoric movements or responses that involve muscle contraction and can be composed of smaller action syllables.

This anatomically serial selection and concurrent engagement is crucial because goals, strategies, and actions require different timescales for planning and execution. While predictive models of varying complexity are necessary for representing goals, strategies, and action sequences^86–89^, we would argue that these loops support both the selection and sustained engagement with these behavioral options. With modular expansion it allows the complexity of these CBG loops to vary between species based on their environmental demands and behavioral repertoire needs^90^. The advantages of nested, hierarchical behavioral automatization becomes particularly evident when pursuing complex goals that require multiple subgoals and actions^73^. This provides a crucial layer of cognitive offloading between high-level planning and action execution. Consciously attending to all of these elements over extended periods would demand significant, maybe at times unattainable cognitive effort. For instance, an experienced driver navigating a familiar route requires less cognitive effort than a novice driving the same route, illustrating the distinction between automatic and conscious control, even when the overall goal remains consciously chosen. Other forms of behavioral automatization, distinct from simple S-R habits, which are not an integral part of the engagement framework suggested here but can be viewed as complementary, have been reported earlier: for instance, “automation of willing”, where frequently pursuing a goal in specific contexts leads to automatic goal activation upon encountering those contexts^91^. Another example is, “goal-dependent habits”, where habitual actions are mentally represented as goal-action links that are automatically triggered only when the associated goal is engaged^92,93^. Both forms of automatization maintain sensitivity to outcome devaluation, reinforcing the principle that automaticity need not eliminate goal-dependence or functional adaptability; and both mechanisms can be combined in a chain with elements of the suggested three-loop engagement framework.

## Separate striatal macro domains support engagement with goals, strategies, and actions

*Which striatal macrodomains support engagement with goals, strategies, and actions? The VS, DMS, DLS* are hypothesized to be the anatomical substrates within the striatum responsible for engagement with *goals, strategies and individual actions* respectively^84,94,95^. The VS may represent goals, the DMS the strategies required to achieve those goals, and the DLS the specific action sequences that comprise a behavioral response (dictated by the currently engaged strategy). Of note, we refer to the rodent macrodomains as the DMS is often considered analogous to the primate caudate nucleus, the rodent DLS to the primate posterior putamen, and the rodent VS to the primate nucleus accumbens^96^. The division of the striatum into three macro-domains is a simplification, other naming conventions based on cortical inputs offer higher anatomical resolution^97,98^. The striatum is characterized by a gradient of cortical and thalamic synaptic inputs shifting from ventromedial to dorsolateral regions^99^. This gradient likely supports engagement with behavioral options across shrinking timescales; from long-term goals in ventro-medial regions to strategies and finally millisecond-scale action syllables in dorso-lateral regions^100^. Despite this, we adhere to the standard three-domain nomenclature in this review for two reasons. First it aligns well with the conceptual distinction between goals, strategies, and actions, and second because the bulk of the existing literature uses this terminology, limiting our ability to map functions to more precise sub-regions.

### Ventral striatum

The proposed role of the VS and the most ventro-medial part of DMS (vmDMS the regions which are strongly innervated by vmPFC projections^101,102^, see also Table-1, projection strength is defined in these anterograde tracing studies based on qualitative metrics of presynaptic fiber and terminal density) in selecting and maintaining engagement with goals provides a unifying framework for many of its classically associated functions. For example, electrical, pharmacological or optogenetic stimulation of the VS in humans and animals enhances incentive salience^103–105^, the motivational wanting attributed to goals and their associated cues, and can induce self-stimulation behavior^106–112^. This increased motivational salience is consistent with sustained goal engagement, as stronger engagement with a specific goal would naturally lead to increased motivation to approach or achieve it. The following reports from human VS DBS subjects illustrate how very specific and complex goals may be activated by stimulating this brain region^109^. “Almost immediately (60 s) after switching the stimulation on, one patient was unable to identify any changes, but spontaneously reported that he realized that he was in Cologne, that he never visited the famous Cologne Cathedral, and he planned on doing this in the immediate future, which he indeed did the day following the operation. A second patient’s immediate (60 s) reaction to stimulation was quite similar; she did not report any acute changes in depressive symptomatology but spontaneously mentioned that she wished to take up bowling again (a favorite pastime activity of hers from 12 years before)”. Unlike in human studies, goals in animal experiments must be inferred from choice behavior. Nevertheless, research has demonstrated that ventral striatum activity is necessary for initiating and re-initiating goal pursuit in rats^113,114^. Furthermore VS activity represents predicted outcomes in rats and primates^115–117^, and predicts or precedes behavioral engagement with goals in rats^103,118,119^.

Such reports further suggest that VS stimulation promotes engagement with specific, long-term goals, not just aspecific reward seeking. Similarly, the VS’s role in addiction^120,121^ can be viewed as a pathological narrowing and intensification of goal engagement by prioritizing a drug-induced outcome state at the expense of other goals. VS lesions impair learning driven by changes in reward value^3^, potentially indicating its role in goal-engagement and in updating goal representations.

Finally, beyond connections to the midbrain dopaminergic system the VS’s connections to the ventral pallidum, substantia nigra pars reticulata, and through them ultimately to multiple PFC regions^122–124^, provide an anatomical substrate for communicating goal engagement to other brain areas, influencing downstream strategy and action selection.

### Dorsomedial striatum

The DMS is proposed to be involved in the selection of, and sustained engagement with, strategies to achieve selected goals. Inactivation of the DMS impairs both reversal learning and strategy switching^125^, demonstrating its critical role in adapting strategies based on feedback. DMS neurons represent action-values^100^ and advantageous strategies^6^ in probabilistic decision-making tasks, suggesting that the DMS encodes information necessary for choosing and maintaining a particular strategic approach. Furthermore, dorsal striatal neurons, including those in the DMS, exhibit firing rate correlations with stimulus-value in classical and instrumental conditioning paradigms^126,127^ indicating a role in learning and representing the value of different sensory cue based strategies. Recent evidence suggests a functional specialization within the DMS along the anterior-posterior axis, with the posterior DMS involved in learning and committing to strategies based on positive outcomes, and the anterior DMS involved in utilizing the lack of expected reward, potentially promoting switching to new strategies^2,128^. Further research is needed to determine whether this anterior-posterior specialization reflects a mechanism for regulating engagement with individual strategies or represents a fundamentally different role for the anterior DMS.

### Dorsolateral striatum

In goal-directed behavior, the DLS is proposed to be responsible for the engagement with actions in the correct order and duration, which can be dictated by a currently engaged rule or strategy^95^. DLS neurons systematically encode information about the identity, timing, duration and ordering of sub-second action syllables that form actions. Lesioning of the DLS prevents appropriate action-syllable-order in sequences^34^. DLS MSN ensembles encode action identity independently of movement speed^33^, suggesting that the DLS represents the selected action itself, rather than how it is being executed. MSN ensemble activity represent actions, in a way that multiple populations of MSNs –including cells which change their activity throughout the duration of commitment^129–131^ and also cells which increase their activity at the onset or offset of it^131,132^ –together determine the time of action engagement. Thus, the DLS, as a whole can be viewed as a switchboard representing the entire space of available actions^33^.

## Matrix and striosome micro-domains support different functions

**Fig. 2:**
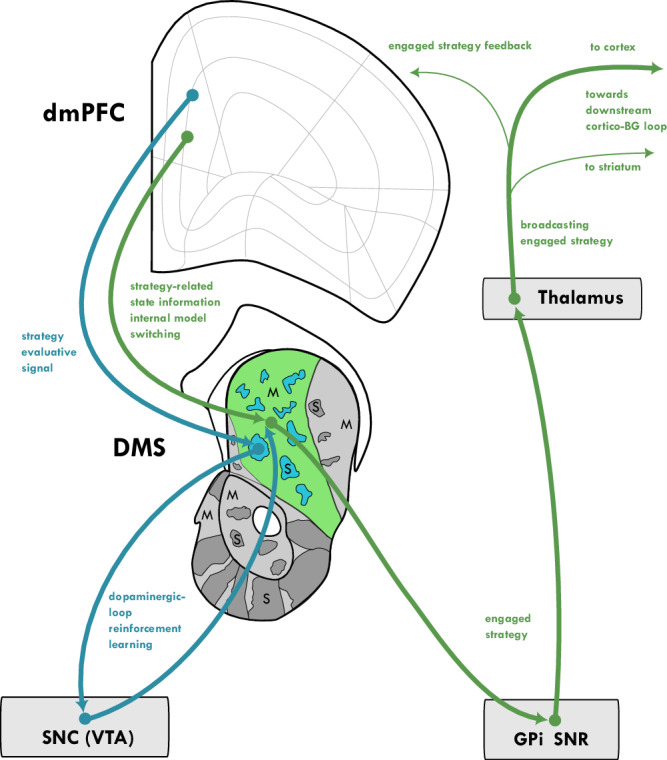
Fast and Slow Prefrontal Modulation of Striatal Strategy Engagement via Matrix and Striosome Pathways in goal-directed behavior. When environmental contingencies are stable, active matrix MSN populations represent the currently engaged strategy. The PFC modulates this engagement through two primary mechanisms: (1) Direct Matrix Modulation: The PFC sends information about the current internal model state directly to matrix MSNs. This input can rapidly alter the active MSN population, leading to a switch in the engaged strategy. (2) Striosome-Mediated Learning: The PFC provides model-based reward prediction information to striosomes. This input, relayed via the dopaminergic system, modulates synaptic plasticity in the matrix, facilitating long-term learning and adaptation of strategy selection. This figure represents the middle hierarchical loop from Fig. 1, strategy selection and engagement; here, only key brain regions involved in probabilistic decision-making are shown (in the absence of sensory cue instructions about strategy). While appearing as patches in 2D sections, striosomes should be conceptualized as a 3D network of pipelines.

What are the hypothesized functions of striatal microdomains in goal-directed behavior? Within each striatal macrodomain, microcompartments known as striosomes (or patches) and the surrounding matrix^20,133–135^ exhibit distinct histochemical properties and connectivity, and thus are hypothesized to serve specialized functional roles (Fig. 2). Only MSNs from the striosome compartment have strong direct projection to the evaluative midbrain dopaminergic cell groups (VTA, SNc), whereas MSNs from matrix project only to non-evaluative BG nuclei (GPe, GPi and SNr)^13,136^, and they communicate more within microcompartment borders than across^21,137,138^. Based on these circuit differences and stronger representation of reward-predictive, expected-outcome-related signals in striosomes compared to matrix^139–141^ we adopt the microdomains based actor-critic framework^142,143^, expected to generalize across the VS, DMS, and DLS macrodomains.

### Matrix

MSN ensemble activity within the matrix compartment is hypothesized to represent the currently engaged behavioral option. Houk, Adams, and Barto proposed that matrix modules function as an actor, which generates signals that command actions or represent plans^142^. In actor-critic architectures, the actor is defined as “a conventional action–selection policy, mapping states to actions in a probabilistic manner“^144^. Similarly, Joel and colleagues suggested that small populations of striatal matrix neurons are capable of eliciting actions^145^, based on the actor model proposed by Suri and colleagues^143,146^. Ito and Doya rephrased the proposal that the matrix compartment, particularly within the dorsal striatum, implements the “actor” that learns action selection^100,142,145^. They further suggested that matrix neurons learn “action values” of candidate actions, contributing to model-based action selection and flexible, goal-directed behavior^147–150^. It is important to acknowledge that the literature supporting the specific role of the matrix compartment in representing engagement with actions are primarily theoretical and computational. To our knowledge, direct experimental evidence specifically isolating the matrix compartment’s contribution to action engagement is currently lacking. However, as Crittenden and Graybiel noted, the matrix comprises 80-85% of the dorsal striatum’s volume, suggesting that the majority of MSNs reside within this compartment^151^. Therefore, it is highly likely that existing data supporting the role of dorsal striatal MSNs in action selection and execution^34,36–38^ predominantly reflect the activity of matrix MSNs. Future research should prioritize directly assessing the specific contributions of matrix and striosome compartments to behavioral engagement and other aspects of goal-directed behavior.

### Striosomes

Striosomes represent evaluative signals about the currently engaged behavioral option. Striosomal neurons fire more to reward-predictive activities, reward-predicting cues and encode more information about expected outcome during learning^139,141^. Further, striosome compartments consistently exhibited stronger representations of reinforcement outcomes than matrix^139^, and mediate value based learning^140^. In line with this evidence one version of the actor-critic model posits that striosomes realize the critic role by integrating and providing reward predictive information (“state value”), and its derivatives like RPE, to the actor^142,145,150,152^, through a dopaminergic loop (see also later sections). In actor-critic architectures, “The critic corresponds to a conventional state-value function, mapping states to expected cumulative future reward“^144^. Whether striosomes only influence matrix-based engagement with behavioral options, through the midbrain dopaminergic cell system and learning and relearning, or whether there are other mechanisms, is an open question and requires more research; there is evidence suggesting that MSNs communicate less across striosome-matrix borders then within^21,137,138^.

An alternative actor-critic model assigns the critic role to the VS or nucleus accumbens, and the actor role to the DS, rather than to striosomes and matrix, respectively^153–155^. However, several lines of evidence support the striosome-matrix division favored by this review. First, the significantly higher striosome/matrix ratio in the VS compared to the DS^21,156,157^, could confound the identification of critic-related activity in the VS and actor-related activity in the DS. Second, a hierarchical engagement framework, as proposed here, benefits from separate critics for goals and strategies. Because even the optimal strategy may not achieve the desired goal on every trial, the predicted value of the strategy will often be lower than the predicted value of the goal. Therefore, independent evaluation of goals (by VS striosomes) and strategies (by DMS striosomes) allows for more precise adaptive learning.

## Different associative mechanisms underlie striatal engagement at different macro-domains

**Fig. 3:**
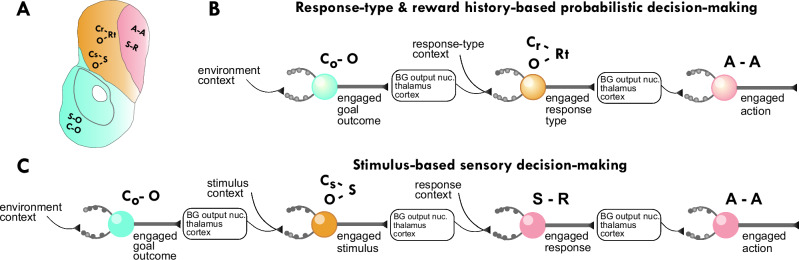
Distinct Associations within Striatal Macrodomains Mediate Engagement with Specific behavioral options. Learning, occurring at dendritic synapses and spines, modifies which inputs drive effectively which MSN ensemble. The activity of these MSN ensembles represents the currently engaged behavioral options, enabling automatization. From a comprehensive list of learned associations, within each striatal macrodomain (**A**); the sequential selection is depicted in (**B**) for response type and reward history based probabilistic decision-making, and in (**C**) for goal-directed decision-making driven by sensory cues.

What associative mechanisms can underlie striatal engagement at different macrodomains? Existing data support a framework in which MSN ensemble activity itself can represent the timing of commitment to behavioral options, in a way that multiple populations of MSNs –including cells which change their activity throughout the duration of commitment^129–131^ and cells which increase their activity at the onset or offset of it^131,132^. Specific actions are represented continuously in neural activity space by overlapping, rather than discrete, MSN ensembles^33^. Striatal circuit architecture enables the emergence of winner-takes-all (WTA) MSN ensembles representing engaged options; these circuit motifs include fast-spiking interneuron-based^158,159^, and MSN lateral inhibition-based suppression of competing MSN ensembles^160,161^, as well as iSPN-mediated feedforward suppression of competing BG-output nuclei ensembles^162^. The WTA ensemble activity is driven by convergent synaptic inputs from various cortical and subcortical regions, among which cortical is the largest and thalamus is the second largest source^163,164^, highlighting the importance of cortico-striatal and thalamo-striatal inputs in determining moment by moment WTA ensembles^66–72^. Convergent inputs combined with synaptic plasticity make the striatum a powerful substrate for associative learning and switching between engaged options^165^. This learning process establishes a mapping between specific input patterns and behavioral options. Within this framework, we define engagement as the instantiation of a learned association, where a specific pattern of synaptic input drives the postsynaptic activity of a corresponding MSN ensemble. Therefore, ‘associative learning’ is the process of creating the input-output map, while ‘engagement’ is the real-time use of that map. With these definitions in mind, we will now examine the associative processes within each striatal macrodomain to elucidate how engagement is realized across the striatum (Fig. 3). The traditional nomenclature, describing associations as stimulus-response (S-R, in DLS), stimulus-outcome (S-O, in VS), or response-outcome (R-O, in DMS), is not consistent from the MSN output point of view for all the macro domains. Especially the R-O association in the DMS, having the outcome as the last element of the association would not explain well the strategy engagement related MSN activity. We suggest that a more biologically grounded approach should emphasize the output of the striatum, the MSN ensemble activity itself. Therefore, we propose a revised nomenclature where the last element in the abbreviation represents the postsynaptic MSN ensemble activity, and the preceding elements represent the presynaptic inputs that drive that activity.

## Ventral striatum: engagement with Goals via S-O and C-O associations

The VS has established roles in representing stimulus-outcome (S-O) associations in both rodents and primates^166,167^, in which sensory stimuli serve as predictors of specific outcomes. We propose to augment this framework by incorporating context-outcome (C-O) associations within the repertoire of VS function. This addition reflects the influence of broader environmental contexts on outcome prediction, enabling a more comprehensive neuronal representation of goal-relevant information within the VS. The activity of VS MSNs represents the outcome (O) – the goal – and this activity is driven by inputs representing either specific stimuli (S) or the broader context (C). For example, VS activity is necessary for initiation of stimulus based engagement with goals in rats^113,114^, and 40% of VS neurons discriminate between different expected rewarding liquid outcomes based on visual cues in primates^168^; this discrimination can also be interpreted as a neuronal representation of engagement with specific goals. Multiple further studies corroborate the role of VS MSNs in encoding the properties of desired outcomes^103,115,116,169–172^, supporting the idea of S-O and C-O associations. Here, an outcome is defined as a specific external event (change in the environment) contingent on a chosen action —for example, a rewarding object (food pellet, coin), a state change of the environment (gate opening), or sensory feedback (auditory cue); whereas a goal is the active, internal representation of that outcome when it is currently being pursued.

## Dorsomedial striatum: engagement with strategies via O-C-S and O-C-RT associations

The DMS is proposed to support engagement with strategies to achieve selected goals. Its role in adapting strategies is demonstrated by deficits in reversal learning and strategy switching following its inactivation^125^. DMS neurons encode information for maintaining a strategic approach, such as action-values in probabilistic tasks and stimulus-values in conditioning paradigms^6,100,126,127^. We argue this is mediated by outcome-context-stimulus (O-C-S) and outcome-context-response type (O-C-RT) associations, which better capture the information processing required for flexible, rule-based behavior than traditional R-O models. In this revised nomenclature, DMS MSNs represent either the key stimulus (S) in sensory-guided tasks or the response type (RT) in probabilistic tasks, driven by inputs conveying the desired outcome (O) and strategy relevant elements of the current context (C). This is supported by findings that stimulus-responsive MSNs are concentrated in the DMS during visual decision-making (the last element in the O-C-S associations), and that DMS neurons represent advantageous response types during probabilistic tasks (the last element in the O-C-RT associations)^6,100,173–175^.

## Dorsolateral striatum: engagement with actions via S-R and A-A associations

Finally, the DLS mediates engagement with actions, controlling their sequence, timing, and duration according to the engaged strategy^95^. DLS neurons systematically encode the identity and temporal structure of action syllables, and represent the selected action itself, independent of its execution speed^33,34^.

This function is consistent with stimulus-response (S-R) associations, where MSN ensemble activity represents action engagement driven by cortical and subcortical stimulus (S) related inputs^66–72^. We further propose that the DLS supports action-action (A-A) associations, a mechanism crucial for chaining actions into skilled sequences. In this scheme, DLS MSN activity representing the current action is driven by synaptic inputs conveying information about the preceding action, a mechanism strongly suggested by the sequential nature of action encoding in DLS neurons^34,36,37^.

This revised associative framework, incorporating a potentially non-exhaustive expanded set of association types, can facilitate a more comprehensive understanding of how striatal subregions contribute to the selection, execution and engagement of goal-directed behaviors.

## Different PFC regions support the selection and engagement with goals, strategies and actions

The functional specialization and preferential striatal projection topology of different PFC regions raises the question, which prefrontal areas support the selection and engagement with goals, strategies, and actions? At the highest level of the associative cortical hierarchy, the PFC plays a crucial role in representing actions (in the broadest sense, behavioral options), a fundamental requirement for organizing goal-directed behavior via mechanisms such as short-term memory and anticipatory sets^176,177^. The PFC provides the neural substrate for executive control and working memory^178–180^; furthermore, it generates goals, predicts outcomes based on environmental context^181–183^ and enables flexible, goal-directed behavior guided by an internal world model^3,30,31,184–187^. Despite the widespread neuronal representation of task-relevant variables throughout the PFC during goal-directed behavior^188–190^, considerable research supports functional specialization within the PFC. This specialization is also evident in the topographic projections from different PFC subdivisions to distinct striatal macrodomains, which suggests a division of labor for selecting goals, strategies, and actions^84^ (see also Table-1, and the Box 1; the latter illustrates, through specific behavioral paradigms, how multiple prefrontal regions, operating within serially connected PFC-BG loops, support the hierarchical selection of behavioral options).

The framework presented here aligns with a unified cortico-thalamic system view rather than treating these structures as separate functional entities, based on their extensive bidirectional connectivity^191,192^, complementary circuit motifs^191^, long-range trans-thalamic cortico-cortical relay function, and broadcasting hub operations^193^. One particularly interesting line of evidence shows that PFC exhibits recurrent excitatory circuit connectivity^194^ and high-dimensional, mixed-selective representations of the environment^195,196^. Complementarily, feedforward circuit motifs (without recurrent-excitation) enable the thalamus to extract abstract, low-dimensional representations from these high-dimensional PFC representations^197–201^, and broadcast this abstracted information to other cortical areas or back to the originating regions. Further evidence suggests that the thalamus may play an even more prominent role in rule representation^202^, based on earlier emergence of abstract rule encoding in thalamus compared to PFC (though it should be noted that only two, not all potential PFC subregions were investigated simultaneously). Further arguments for integrative cortico-thalamic function are findings on communication through coherence^203–205^, where the thalamus can play a pivotal role in orchestrating such coherence^206–210^. Therefore, in this review thalamus is viewed as part of an integrated cortico-thalamic system. Thalamic relaying of prefrontal computations to striatum has also been suggested^211^; however, striatum itself also exhibits circuit motifs without recurrent excitation^159,212^ and has been suggested to perform dimensionality reduction from cortical representations^145^.

BOX 1 Applying the hierarchical engagement framework to specific behavioral paradigms *A) Attentional set-shifting paradigm*^325–328^ *(Rodent example)*: In this paradigm, the subject must learn rules based on sensory dimensions (e.g., odor or medium) to obtain a single outcome type (food). *Loop 1 (Goal)*: The vmPFC/OFC computes the environmental context and valuation of the potential outcome. VS ensemble activity maintains engagement with the goal and thus motivation for the task. *Loop 2 (Strategy)*: mPFC/OFC predicts/tracks the subject’s position in task-state-space based on an internal world model, detects task-state changes and strategy/set-shifts (e.g., “Odor A is no longer rewarded; switch to digging medium A”); and in turn it biases the DMS to engage the currently advantageous strategy (e.g., “Focus on odor”). DMS ensembles represent the engagement with this specific rule or attentional set. *Loop 3 (Action)*: Motor cortices and adjacent A24 compute the current advantageous response based on the engaged strategy and current trial cues (e.g., “Strategy says odor A is correct; odor A is on the left; therefore, go Left”). The DLS maintains engagement with this chosen motor response and its constituent action syllables. (Note: In these cited studies, functional evidence is primarily derived from lesion effects in mPFC/OFC and DMS. The specific contributions of VS and DLS to this paradigm are inferred from the wider literature; see main text)

*B) Probabilistic Choice / Bandit Tasks*^6,84,222^ *(Rodent & Primate example)*: Subjects choose between multiple options (e.g., 2 or 3 ports/targets) where reward probabilities change over time, requiring continuous value updating. *Loop 1 (Goal)*: The vmPFC/OFC computes the expected value of the chosen option. VS ensemble activity encodes and maintains this value signal throughout the trial, representing a temporal commitment (engagement) to the goal that drives continuous motivation. *Loop 2 (Strategy)*: The mPFC tracks the action-outcome history (internal model) to detect changes in reward contingencies; and in turn it biases the DMS to engage the currently advantageous strategy (e.g., “Shift preference to Left”). DMS ensembles encode the specific action-values (e.g., Value of Left vs. Right) required to select the optimal strategy. *Loop 3 (Action)*: Motor cortices and lateral PFC compute the immediate motor kinematics. DLS/DS activity represents engagement with the specific execution of the chosen movement (e.g., reach direction or port choice), emerging later in the trial than VS goal signals. (Note: This entry synthesizes findings across species: Primate studies^84^ provide direct evidence for the dissociation of Goal (VS) and Action (DS) engagement, while rodent studies^6,222^ elucidate the Strategy/Action-Value updating in the mPFC-DMS loop.)

*C) Economic decision-making, goods-based*^215,216,329^ *(Primate example)*: In this paradigm, in each session there are two predicted appetitive outcome types (fruit juices, offered in different quantities). *Loop 1 (Goal)*: vmPFC/OFC computes environmental context and sensory-cue-based prediction, valuation, and comparison of potential outcomes/goals; VS ensemble activity represents engagement with the chosen goal for each trial (e.g., choose 2 × 65 µL of grape juice over 3 × 65 µL of diluted cranberry juice). *Loop 2 (Stimulus-selection)*: mPFC/OFC maintains the chosen-value signal and outcome identity, and sends this information to bias DMS toward the sensory cue associated with the desired outcome; based on the engaged goal, DMS ensemble activity represents engagement with the sensory cue associated with the desired outcome (e.g., bright green squares for grape juice). *Loop 3 (Action)*: Motor cortices and adjacent A24 compute the current advantageous response based on the engaged sensory cue and current trial spatial configuration (e.g., engaged sensory cue is bright green squares, and bright green squares are on the left side, therefore choose the left-side response), and send this information to bias DLS toward the advantageous response; based on the engaged sensory cue, DLS ensemble activity represents engagement with the current trial’s chosen response according to the current trial cues (e.g., choose the left side as the left cue—bright green squares—is the one associated with the desired outcome), and in subsequent hierarchical sub-loops, the moment-by-moment engagement with the current action-syllable within the engaged response. (Functional evidence was obtained from OFC via electrophysiology and optogenetics; but VS, DMS, DLS, mPFC, and motor cortex/A24 were not recorded in the cited studies, so the goal-engagement (Loop 1 striatal component), stimulus-selection (Loop 2), and response-execution (Loop 3) stages of the framework remain to be directly tested in this paradigm, and have separate support from other studies, see main text.)

*D) Combined fixed- and free-choice probabilistic decision-making*^100^ *(Rodent example)*: In the fixed-choice task, specific frequency tones instructed the rats to poke a designated left or right hole for a fixed probability of reward. In contrast, the free-choice task utilized a white noise cue that provided no directional information, requiring the rats to select a side based on reward probabilities that varied across blocks. *Loop 1 (Goal)*: VS ensemble activity showed the strongest activation at task initiation, encoding state value and task type (fixed vs. free-choice context). This represents engagement with the goal of obtaining reward and the general incentive value of the current behavioral context—setting the stage for downstream action selection. *Loop 2 (Strategy)*: DMS ensemble activity most strongly encoded action values during the action selection epoch—the expected value of choosing left versus right given current reward contingencies; DMS action-value encoding was more prominent during free-choice trials (when the animal must compute and compare values) than fixed-choice trials (when the cue directly instructs the response), consistent with DMS biasing selection toward the currently advantageous choice based on the engaged goal. *Loop 3 (Action):* DLS activity encodes the action command (Left vs. Right identity), peaking just before execution. This signal is prominent even when choices are repeated, consistent with the DLS maintaining the specific motor engagement. (Note: Functional recordings were obtained from all three striatal macrodomains (VS, DMS, and DLS) that provide direct evidence for the hierarchical gradient across striatal stations. PFC regions were not recorded, so the cortical contributions to each loop remain to be directly tested in this task, and have separate support from other studies, see main text.)

### Goal selection

The ventro-medial prefrontal cortex (vmPFC), including the medial OFC, ventral-PrL-, IL-, and frontal pole- cortex (with some interspecies differences) are implicated in goal selection and engagement. For example, Holton and Kolling demonstrated that the vmPFC supports goal commitment in human subjects^213^. The medial OFC plays a critical role in goal selection based on value comparisons, potentially by transforming representations of value into a common currency to facilitate comparisons among diverse options^214–216^. The vmPFC is crucial when affective responses are influenced by conceptual information about specific outcomes^188,217^. This ventromedial region of the PFC works closely together with the VS in the process of goal selection, as evidenced by anatomical connectivity^97,101,102^ and can provide outcome-predictive contextual (C) information that drives VS MSN activity in the proposed C-O associations and goal engagement. Furthermore, striosome-biased projections from these vmPFC regions to the VS (further discussed later) are hypothesized to provide evaluative feedback, influencing striatal learning and updating of goal representations. Also based on their functional data Tang and Averbeck proposed that learned value information representing engaged behavioral goals is maintained throughout the ventral cortico-striatal circuit, which includes the OFC and VS^84^ (see also Box 1, example C).

### Strategy selection

Strategy selection associated parts of the PFC include dorsal PrL-, lateral/ventral OFC-, and ACC cortices^2,4,218^. Ventral/Lateral OFC is associated with sensory cue based prediction of outcomes and outcome-properties, and in turn in cue-based strategies^181,182,219^; whereas mPFC has been linked more with choosing actions based on outcome history^181,220^. Activity of PrL neurons encodes subsequent trial choice during outcome evaluation periods, and optogenetic inactivation of PrL cortex impairs optimal outcome-probability-based decision-making^221^. mPFC neurons persistently encode probabilistic value based decision variables^222^. Both mPFC and dorso-medial striatal neurons represent advantageous strategy (response-type) in a probabilistic decision-making task, a small proportion of those were putative monosynaptic connected mPFC-DMS neuron pairs^6^. The mPFC is also important in the case of complex rules and strategies which can often be considered as internal model based decision-making^30,223,224^. Lateral/ventral OFC is important in various forms of sensory cue based goal-directed behavior^3,31,218,225^. OFC projections to the DMS are required for appropriate economic decision-making^1^ (see also the Box 1 example A and B).

### Action selection

Higher-order motor cortices, including secondary motor cortex and likely the neighboring dorsal part of the ACC (dorsal area 24, ACAd), are primarily associated with selecting and planning upcoming actions^85,226–229^. These regions, in turn, preferentially target the DLS to finalize selection and time the engagement with specific actions. In general, due to methodological constraints, most studies have only recorded and analyzed neuronal responses in one or a few adjacent frontal regions during the same behavior. Only recent improvements in methodology and analysis have begun to enable investigation into differences in information representation across larger sets of regions^230^. This will refine our understanding of inter-regional differences with respect to goal-directed behavior.

## Different PFC inputs drive striatal micro-compartments

**Fig. 4:**
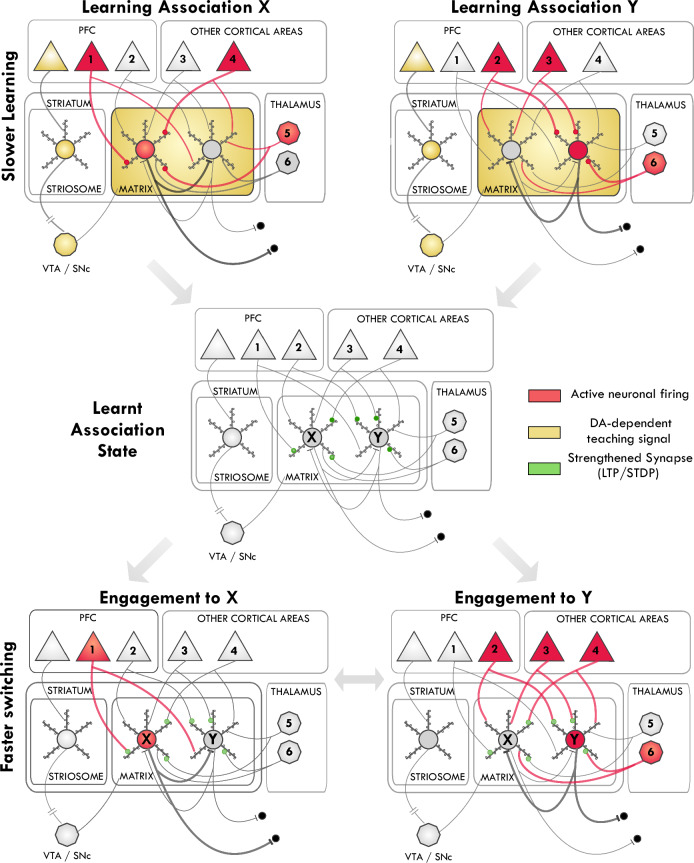
Circuit Model for Prefrontal Modulation of Striatal Engagement through Learning and Switching. In goal-directed behavior PFC modulates striatal function through two primary mechanisms: striosome-mediated slower learning (*Top and Middle rows*) and faster switching through direct matrix modulation (*Bottom row*) once relevant associations are represented. Striosome-Mediated Learning (*Top and Middle rows*): Learning is governed by a three-factor rule based on spike time dependent plasticity: synaptic connections onto matrix MSNs are strengthened when presynaptic input, postsynaptic MSN firing, and striosome-mediated phasic DA (yellow) release (representing a positive RPE) occur concurrently. (note: The mechanisms may differ between D1- and D2-receptor expressing MSNs). (*Top Panels*)**:** The process of learning two distinct associations is shown. On the left, inputs from PFC/thalamic ensembles 1, 4, and 5 drive activity in matrix MSN ensemble X during a DA-dependent teaching signal, initiating plasticity. On the right, a different input pattern from ensembles 2, 3, and 6 drives another MSN ensemble Y, initiating  learning of a separate association Y. (*Middle Panel*): The “Learnt Association State” depicts the result of the learning processes. Enlarged green dendritic spines represent the strengthened synaptic connections (LTP/STDP), establishing a new input-output mapping, or “striatal routing.” Direct Matrix Modulation and Behavioral Switching (*Bottom pane*l)**:** Once associations are learned, the PFC can rapidly switch between them by altering direct input to the matrix. (*Bottom Left*): A specific behavioral option “element of the extended behavioral-space” is engaged as input from PFC ensemble 1 selectively activates the now-potentiated MSN ensemble X. (*Bottom Right*): To switch outputs, a different pattern of cortical/thalamic input (ensembles 2, 3, and 6) activates MSN ensemble Y. Although ensemble 4 provides competing input favoring ensemble X, the stronger convergent input to ensemble Y allows it to win the competition. This winner-take-all process, facilitated by lateral inhibition between MSN ensembles, ensures the selection of a single, dominant behavioral option. This illustrates how dynamic changes in cortical and thalamic input can rapidly shift engagement between established behavioral representations based on the learned synaptic strength profile.

What PFC inputs drive striatal micro-compartments? The PFC likely influences striatal engagement through two primary pathways, mirroring the functional distinction between striosomes and matrix. First, striosome-mediated learning allows the PFC to shape striatal associations –routing of specific inputs to specific outputs– over time by providing evaluative feedback (Fig. 4A, B). Second, direct matrix modulation allows the PFC –and other cortical and thalamic inputs– to rapidly switch between engaged behavioral options by altering the pattern of activity in matrix MSNs (Fig. 4C). These two pathways provide complementary mechanisms for both long-term adaptation and short-term flexibility.

### Striosome-mediated learning pathway

Specific PFC regions project preferentially to striosomes within specific striatal macrodomains, providing evaluative signals that drive learning and adaptation. These signals likely represent model-based reward prediction information—that is, predictions about future outcomes based on an internal model of the environment, rather than simply on past rewards^100^. Such a pathway allows the PFC to influence which associations are learned and strengthened within the striatum, shaping long-term behavioral biases.

Observations of striosome-biased connectivity from distinct PFC regions suggest an organization which can support functional specialization. Specifically, a striosome bias from a particular PFC region to a given striatal macrodomain may only be present where the respective PFC region provides critical evaluative signals. Projections from the same PFC region to other striatal areas, where the evaluative role of that PFC region is not critical, may not exhibit striosome bias.

Supporting this concept, Ragsdale and Graybiel found that fronto-striatal projections correspond to a large extent with striatal microdomains^231^, more specifically PrL, parts of ACC and posterior OFC send dense innervation to striosomes in the dorsal striatum, with less dense labeling in the surrounding matrix^232,233^. Injections into motor cortices and parts of cingulate cortex showed preferential termination in the matrix^232^.

In more detail multiple tract-tracing studies demonstrated that cortical regions with striosome-biased striatal connectivity do not project to all parts of the striatum in the same striosome-biased way; rather only to certain areas of the striatum^232,233^. Similarly, Waugh and Blood found that striosome-like voxels with connectivity dominated by one cortical region are organized into distinct zones^234^.

Ragsdale and Graybiel observed a similar organizational principle in cats, where corticostriatal projection patterns could be ordered along a single axis according to the dorso-ventral position at which the transition from striosome-biased to striosome-avoidance occurred^235^. This axis included projections from the posterior parietal cortex, dorsomedial PFC, vmPFC, insular cortex, and rostral temporal cortex. In combination, the observed striosome bias in corticostriatal projections can reflect a functional organization, where different cortical regions contribute distinct evaluative information to specific striatal domains.

The medial OFC, IL cortex, and ventral parts of the PrL cortex strongly project to the nucleus accumbens of the VS^97,101,102^. These brain structures have been associated with functions consistent with goal selection, suggesting that striosome bias from these cortical areas may be present primarily in striatal macrodomains involved in goal selection and subsequent strategy selection, such as the VS, but not in the DMS and DLS. Similarly, the dorsal PrL cortex and ventral ACC strongly project to the DMS^102^. These regions have been associated with functions consistent with strategy selection, suggesting that striosome bias from these cortical areas may be present primarily in striatal areas involved in strategy selection, such as the DMS, but not in the nucleus accumbens or DLS. Finally motor cortices (M1 and M2) and dorsal ACC strongly project to the DLS^98,101,102^. These regions have been associated with functions consistent with action selection^33,34^, suggesting that striosome bias from these cortical areas may be present primarily in striatal areas involved in action selection, such as the DLS, but not in the nucleus accumbens or DMS.

### Direct matrix modulation

In addition to the slower, learning-based influence via striosomes, the PFC can also exert direct control over striatal activity through projections to matrix MSNs^14^ (Fig. 4C) or connections to striatal interneurons^55^. These projections can convey information about the current state of the internal model, allowing the PFC to rapidly switch between engaged behavioral options^3,30,31,56,57^. This rapid modulation is critical for flexibly adapting behavior based on the current state of an internal model, without waiting for slower, dopamine-dependent plasticity. For instance, projections from motor cortices (M1/M2) to the DLS matrix are well-positioned to directly influence the selection and engagement with actions^85,226–228^, while projections from associative PFC regions to the DMS matrix could rapidly update an engaged strategy based on new contextual information^3,30,31^. Importantly, the transition between engaged options does not only rely on PFC as a central controller. Instead, switching is an emergent property of prefronto-BG systems cooperation, where PFC provides internal-world-model state-based predictions as biasing signals about best behavioral options to striatum which executes winner-takes-all selection and engagement of best option^212,236^. When state-transition is detected and best option predictive biasing signals from PFC are changed, the input drive for the active matrix MSN ensemble decreases, destabilizing the current engagement. This allows alternative options, driven by new contextual PFC inputs, to overcome lateral inhibition and emerge as the new dominant option.

The capacity to maintain engagement with a goal or strategy is constrained by the cognitive cost of the underlying internal model. Working memory processes in the PFC provide the top-down ‘context’ signals required to sustain activity in specific matrix ensembles. Critically, the capacity to generate and maintain these context-dependent biasing signals is limited by working memory resources. When cognitive load is high—such as during dual-task conditions, complex planning, or situations requiring the simultaneous maintenance of multiple task-relevant representations—the fidelity and stability of PFC-to-matrix signals may degrade^237,238^. This degradation reduces the system’s ability to sustain goal-directed engagement and can cause the balance of behavioral control to shift toward previously reinforced, habitual response patterns that are less dependent on active PFC maintenance^239,240^. Thus, the direct matrix pathway represents not only a mechanism for flexible, context-sensitive switching, but also a point of vulnerability where resource limitations can compromise goal-directed control in favor of more automatic, stimulus-driven responding.

## Striosomes exert their evaluative teaching function through a mid-brain dopaminergic loop

How do striosomes exert their evaluative teaching function within the striatum? Projections from striosomes are the major source of striatal input to VTA/SNc^136^ and provide a dopaminergic mechanism for evaluating ongoing behavior and updating behavioral option selection in the neighboring matrix compartment. This feedback loop enables the striatum to learn and adapt, ultimately refining the selection of appropriate behavioral options in different contexts.

Striosomes exert their evaluative influence primarily through projections to dopaminergic neurons in the VTA and SNc^136^. These dopaminergic neurons, in turn, project back to the striatum, creating a loop that serves as the basis for modulating engaged behavioral options (represented in the matrix), through changing synaptic weights to learn or relearn (Figs. 2 and 4). Different striatal regions project topographically to distinct areas within the midbrain dopaminergic system, and the dominant input to these dopaminergic neurons dictates their principal return projections to striatum, creating multiple parallel loops that facilitate learning within each striatal macrodomain^76,78,124,241,242^. Alongside these topographically defined “closed” loops, hierarchical striato-nigral projection patterns also allow for a cascading dopaminergic influence from ventral towards dorsolateral striatal regions, enabling cross-domain communication^76,241,243^.

Striosome direct pathway neurons send numerous collateral projections to the GP, SNr, and the SNc. A key distinction between striosome and matrix direct pathway neurons is their projection targets; while both project to the SNr, only striosome neurons directly innervate the SNc^13^. This direct innervation of the SNc by striosomes has been further confirmed using Cre-dependent GFP expression in patch-Cre mice (Sepw1-NP67)^14^. Evans and Khaliq observed a high density of striosome-originating synaptic terminals on SNc dendrites that extend into the SNr^48^. These findings suggest that striosomes exert a powerful inhibitory influence on SNc dopamine (DA) neurons, followed by a period of post-inhibition excitation^48^. In vivo optogenetic studies demonstrate the functional consequences of this influence: activating striosomal projections to the SNc suppresses DA release^53,244,245^, and modulates habit formation^53^ and movement^245^. Importantly, striosomes can also increase DA neuron activity via two reported mechanisms. The first is a polysynaptic pathway via the globus pallidus and lateral habenula^246,247^. The second, originating from striosomal D2R-expressing neurons, involves a single inhibitory relay in the globus pallidus externa^248^. This demonstrates that striosomal direct and indirect pathway neurons can bidirectionally modulate DA release.

This dopaminergic feedback, originating from striosomal input, drives long-term synaptic plasticity at cortico-striatal synapses^52^, shaping which inputs ultimately trigger the engagement with specific behavioral options represented in the matrix. Strong, phasic DA release potentiates concurrently active cortico-striatal synapses onto active MSNs (long term potentiation through spike time dependent plasticity), an effect dependent on D1 receptor activation^49–51,249–252^.

Although this review emphasizes the contribution of DA to striatal learning, and the role of cortico-striatal and thalamo-striatal inputs in fast switching; the multifaceted nature of dopaminergic signaling likely allows for more functions. Baseline dopamine through D1 receptors increases excitability of dSPNs^253^, which can have a permissive effect on selection and engagement of behavioral options^254^. Even though fluctuations in striatal DA, characterized by varying temporal dynamics (including baseline DA levels, phasic, and tonic release), are suggested to be associated with diverse functions, including motor control, action initiation, movement vigor and motivation^255–258^; both phasic and slower ramping dopamine appear to provide reward prediction error signals for optimized adaptive processes^259^. While DA also exerts immediate short lasting effects on cortico-striatal synaptic strength^260^ and modulates the timing of MSN excitability^261^ thus sharpening the population of active MSNs, potentially fine-tuning striatal engagement; the primary drivers of striatal engagement are the direct cortico-striatal and thalamo-striatal inputs as they define which specific populations of matrix MSNs are being activated. Evidence suggests that DA signal kinetics and synaptic plasticity mechanisms differ between the DMS and DLS^262,263^, and between dorsal and ventral striatum^264^. Wave-like DA dynamics could set model expectations and allow early prediction of upcoming behavioral-output adaptations^262^, potentially under direct cortical input influence. However, it remains an open question what diverse functions DA signals support in striatum beyond learning^265,266^. In general, within our framework, one would predict a gradient of slower temporal dynamics in ventral versus dorsolateral striatal regions. Such a gradient would align with the different computational demands of each region—slower integration for goal-related updating versus faster processing for action selection—and is consistent with recent findings^264^.

## Multiple evaluative brain systems exhibit striosome-biased projections for integrative control over striatal learning and, in turn, engagement

Why do multiple evaluative brain systems exhibit biased projections towards striosomes? The biased projection of multiple evaluative brain systems to striosomes suggests a crucial role for this microdomain in integrating diverse value signals to guide adaptive learning and behavioral updates. This convergence of information allows the striatum to create a comprehensive representation of the overall value associated with currently engaged and potential behavioral options, considering not just immediate reward, but also long-term goals, emotional context, and potential risks. Human MRI connectivity data presents a striosome-biased input, revealing preferential projections from several PFC regions (frontal pole cortex, medial frontal cortex, OFC, ACC), insular cortex, amygdala, and mediodorsal thalamus^234^. In contrast, the matrix receives a larger proportion of inputs from secondary motor cortex and visual cortex^14^, reflecting its role in representing and executing specific actions and strategies. Multiple brain regions involved in value and valence processing send striosome-biased projections to the striatum, as shown by anatomical tracing studies. These include the mPFC^232^, OFC^233^, amygdala^267^, BNST, hypothalamus, and anterior and reticular thalamic nuclei^14^. Notably, several of these subcortical inputs—particularly the amygdala, BNST, and hypothalamus—are critically involved in processing threat, risk, and emotional salience, suggesting that striosomes serve as a convergence point where potential dangers and aversive outcomes are integrated alongside reward-related information. We hypothesize that the convergence of diverse evaluative inputs onto striosomes allows for a weighted integration of these signals. This integrated value signal, relayed via the striato-nigro-striatal pathway, modulates DA release to drive synaptic plasticity in the matrix, thereby updating the associations responsible for engaging with specific goals, strategies, and actions. Further evidence for this integrative evaluative function comes from distinct dopamine kinetics; unlike in the matrix where aversive cues decrease DA concentration, striosomal DA release is elicited by cues predicting both rewards and aversive outcomes^268^. This bidirectional sensitivity to valence suggests that striosomes are specialized for processing a broad spectrum of motivationally relevant information—including potential risks and threats—enabling the system to update behavioral policies based on both appetitive opportunities and aversive contingencies.

Importantly, the balance of influence between these different inputs to striosomes can shift. Under conditions of heightened emotional arousal, perceived threat, or high reward-punishment conflict, the influence of subcortical structures such as the amygdala and hypothalamus on striosomal activity may become more pronounced relative to the PFC^46,269,270^. This shift can bias the integrated evaluative signal toward risk-averse or emotionally-driven behavioral patterns. For instance, when potential negative outcomes are salient, increased amygdala drive to striosomes may promote learning that favors avoidance or cautious strategies, even when PFC-based cost-benefit analyses might favor approach. In pathological conditions characterized by chronic stress, anxiety, or trauma, this imbalance may become entrenched, potentially leading to maladaptive avoidance behaviors or impulsive reactions driven by threat-detection systems rather than deliberative evaluation. More research is required to determine whether integrative or winner-takes-all mechanisms are present at striosomes at different homeostatic and emotional states. The influence of subcortical evaluative inputs on striosomes is expected to decrease along the ventro-medial to dorso-lateral striatal axis, reflecting a presumed stronger role of emotions and innate drives in goal selection compared to selecting action syllables.

## Conclusion and outlook

This conceptual review synthesizes a framework for understanding prefronto-striatal interactions in goal-directed behavior, centered on the concept of hierarchical engagement (Fig. 1). We argue that the striatum functions as a “switchboard”, selecting and maintaining engagement with behavioral options (goals, strategies, and actions) under the modulatory influence of the PFC. VS, DMS, and DLS are proposed to mediate engagement with goals, strategies, and actions, respectively. We suggest a revised associative framework (for instance O-C-S and O-C-RT associations in the DMS, Fig. 3), which may more accurately represent the information flow from synaptic inputs to MSN outputs, for specific behavioral engagement types. The PFC influences striatal engagement through two main pathways: striosome-mediated learning, which shapes long-term associations via evaluative feedback, and direct matrix modulation, which enables rapid switching between engaged behavioral options (Figs. 2 and 4). Striosomes, receiving input from multiple evaluative brain systems, integrate diverse valence signals to guide decision-making. This hierarchical engagement framework provides a perspective on prefronto-striatal function and offers new avenues for investigating the neural basis of goal-directed behavior.

While numerous questions remain at the forefront of PFC and basal ganglia research, one critical task is to determine the precise mechanisms by which the PFC modulates striatal activity through both striosome-mediated learning and direct matrix projections. Including, how information about the identity of and transition between different states of the internal world model is transmitted from the PFC to different striatal cell types. This will require further refinement and combination of methods for the simultaneous recording and manipulation of activity in specific PFC and striatal subregions, including striatal microdomains, during well-defined behavioral tasks. Alongside, the accurate location tracking and reporting of functional data will help to better delineate functional gradients or borders across regions and subregions. An interesting question also concerns the functional relevance for cortico-striatal projections of Area 24 as it overlaps with projection patterns of several PFC areas particularly in primates. Advanced computational modeling will be essential for integrating these data and testing hypotheses about the dynamic interactions within prefronto-striatal circuits. How other evaluative regions like the amygdala, habenula, BNST or hypothalamus compete with prefrontal inputs to modulate goal-directed behaviors, remains less understood, and requires substantial new efforts. Finally, the investigation across these levels on how these circuits are disrupted in disorders of goal-directed behavior (e.g., addiction, OCD, schizophrenia, depression) will not only provide critical insights into the pathophysiology of these conditions but also identify potential therapeutic targets. A deeper understanding of prefronto-striatal engagement promises to advance both fundamental neuroscience and the treatment of debilitating neuropsychiatric disorders.

## Supplementary information

Transparent Peer Review file

## References

[1] Gore, F. et al. Orbitofrontal cortex control of striatum leads economic decision-making. *Nat. Neurosci.* **26**, 1566–1574 (2023). 37592039 10.1038/s41593-023-01409-1 PMC10471500

[2] Hart, G., Bradfield, L. A., Fok, S. Y., Chieng, B. & Balleine, B. W. The Bilateral Prefronto-striatal Pathway Is Necessary for Learning New Goal-Directed Actions. *Curr. Biol.* **28**, 2218–2229.e2217 (2018). 30056856 10.1016/j.cub.2018.05.028

[3] McDannald, M. A., Lucantonio, F., Burke, K. A., Niv, Y. & Schoenbaum, G. Ventral striatum and orbitofrontal cortex are both required for model-based, but not model-free, reinforcement learning. *J. Neurosci.* **31**, 2700–2705 (2011). 21325538 10.1523/JNEUROSCI.5499-10.2011 PMC3079289

[4] Nakayama, H., Ibanez-Tallon, I. & Heintz, N. Cell-type-specific contributions of medial prefrontal neurons to flexible behaviors. *J. Neurosci.* **38**, 4490–4504 (2018). 29650697 10.1523/JNEUROSCI.3537-17.2018 PMC5943977

[5] de Kloet, S. F. et al. Bi-directional regulation of cognitive control by distinct prefrontal cortical output neurons to thalamus and striatum. *Nat. Commun.* **12**, 1994 (2021). 33790281 10.1038/s41467-021-22260-7 PMC8012364

[6] Kőszeghy, Á et al. Medial prefrontal cortex activity precedes dorsomedial striatum in need for change during history-based flexible behavior. *iScience* **28**, 113913 (2025). 41321636 10.1016/j.isci.2025.113913 PMC12662081

[7] Laubach, M., Amarante, L. M., Swanson, K. & White, S. R. What, if anything, is rodent prefrontal cortex? *eNeuro* **5**, ENEURO.0315-18 (2018). 10.1523/ENEURO.0315-18.2018 PMC6220587 30406193

[8] Ongür, D. & Price, J. L. The organization of networks within the orbital and medial prefrontal cortex of rats, monkeys and humans. *Cereb. Cortex* **10**, 206–219 (2000). 10731217 10.1093/cercor/10.3.206

[9] Preuss, T. M. & Wise, S. P. Evolution of prefrontal cortex. *Neuropsychopharmacology* **47**, 3–19 (2022). 34363014 10.1038/s41386-021-01076-5 PMC8617185

[10] Cruz, B. F. et al. Action suppression reveals opponent parallel control via striatal circuits. *Nature* **607**, 521–526 (2022). 35794480 10.1038/s41586-022-04894-9

[11] Tang, S., Cui, L., Pan, J. & Xu, N. L. Dynamic ensemble balance in direct- and indirect-pathway striatal projection neurons underlying decision-related action selection. *Cell Rep.* **43**, 114726 (2024). 39276352 10.1016/j.celrep.2024.114726

[12] Frank, M. J. Adaptive cost-benefit control fueled by striatal dopamine. *Annu Rev. Neurosci.* **48**, 1–22 (2025). 39813735 10.1146/annurev-neuro-112723-025228

[13] Fujiyama, F. et al. Exclusive and common targets of neostriatofugal projections of rat striosome neurons: a single neuron-tracing study using a viral vector. *Eur. J. Neurosci.* **33**, 668–677 (2011). 21314848 10.1111/j.1460-9568.2010.07564.x

[14] Smith, J. B. et al. Genetic-based dissection unveils the inputs and outputs of striatal patch and matrix compartments. *Neuron* **91**, 1069–1084 (2016). 27568516 10.1016/j.neuron.2016.07.046 PMC5017922

[15] Gerfen, C. R. Molecular effects of dopamine on striatal-projection pathways. *Trends Neurosci.* **23**, S64–S70 (2000). 11052222 10.1016/s1471-1931(00)00019-7

[16] Gerfen, C. R. Segregation of D1 and D2 dopamine receptors in the striatal direct and indirect pathways: An historical perspective. *Front. Synaptic Neurosci.* **14**, 1002960 (2022). 36741471 10.3389/fnsyn.2022.1002960 PMC9892636

[17] Gerfen, C. R. & Surmeier, D. J. Modulation of striatal projection systems by dopamine. *Annu Rev. Neurosci.* **34**, 441–466 (2011). 21469956 10.1146/annurev-neuro-061010-113641 PMC3487690

[18] Calabresi, P., Picconi, B., Tozzi, A., Ghiglieri, V. & Di Filippo, M. Direct and indirect pathways of basal ganglia: a critical reappraisal. *Nat. Neurosci.* **17**, 1022–1030 (2014). 25065439 10.1038/nn.3743

[19] Cox, J. & Witten, I. B. Striatal circuits for reward learning and decision-making. *Nat. Rev. Neurosci.* **20**, 482–494 (2019). 31171839 10.1038/s41583-019-0189-2 PMC7231228

[20] Brimblecombe, K. R. & Cragg, S. J. The striosome and matrix compartments of the striatum: a path through the labyrinth from neurochemistry toward function. *ACS Chem. Neurosci.* **8**, 235–242 (2017). 27977131 10.1021/acschemneuro.6b00333

[21] Castro, D. C. & Bruchas, M. R. A motivational and neuropeptidergic hub: anatomical and functional diversity within the nucleus accumbens shell. *Neuron* **102**, 529–552 (2019). 31071288 10.1016/j.neuron.2019.03.003 PMC6528838

[22] Wessel, J. R., Diesburg, D. A., Chalkley, N. H. & Greenlee, J. D. W. A causal role for the human subthalamic nucleus in non-selective cortico-motor inhibition. *Curr. Biol.* **32**, 3785–3791.e3783 (2022). 35841891 10.1016/j.cub.2022.06.067 PMC9511894

[23] Aron, A. R., Herz, D. M., Brown, P., Forstmann, B. U. & Zaghloul, K. Frontosubthalamic circuits for control of action and cognition. *J. Neurosci.* **36**, 11489–11495 (2016). 27911752 10.1523/JNEUROSCI.2348-16.2016 PMC5125216

[24] Hardman, C. D. et al. Comparison of the basal ganglia in rats, marmosets, macaques, baboons, and humans: volume and neuronal number for the output, internal relay, and striatal modulating nuclei. *J. Comp. Neurol.* **445**, 238–255 (2002). 11920704 10.1002/cne.10165

[25] Kuroda, M., Yokofujita, J. & Murakami, K. An ultrastructural study of the neural circuit between the prefrontal cortex and the mediodorsal nucleus of the thalamus. *Prog. Neurobiol.* **54**, 417–458 (1998). 9522395 10.1016/s0301-0082(97)00070-1

[26] Middleton, F. A. & Strick, P. L. Basal-ganglia ‘projections’ to the prefrontal cortex of the primate. *Cereb. Cortex* **12**, 926–935 (2002). 12183392 10.1093/cercor/12.9.926

[27] Dickinson, A. & Balleine, B. Motivational control of goal-directed action. *Anim. Learn. Behav.* **22**, 1–18 (1994).

[28] Balleine, B. W. & Dickinson, A. Goal-directed instrumental action: contingency and incentive learning and their cortical substrates. *Neuropharmacology* **37**, 407–419 (1998). 9704982 10.1016/s0028-3908(98)00033-1

[29] Pezzulo, G., van der Meer, M. A., Lansink, C. S. & Pennartz, C. M. Internally generated sequences in learning and executing goal-directed behavior. *Trends Cogn. Sci.* **18**, 647–657 (2014). 25156191 10.1016/j.tics.2014.06.011

[30] Sharpe, M. J. et al. An Integrated Model of Action Selection: Distinct Modes of Cortical Control of Striatal Decision Making. *Annu Rev. Psychol.* **70**, 53–76 (2019). 30260745 10.1146/annurev-psych-010418-102824 PMC9333553

[31] Knudsen, E. B. & Wallis, J. D. Taking stock of value in the orbitofrontal cortex. *Nat. Rev. Neurosci.* **23**, 428–438 (2022). 35468999 10.1038/s41583-022-00589-2 PMC10511019

[32] Sutton, R. S., Precup, D. & Singh, S. Between MDPs and semi-MDPs: a framework for temporal abstraction in reinforcement learning. *Artif. Intell.-Amst.* **112**, 181–211 (1999).

[33] Klaus, A. et al. The spatiotemporal organization of the striatum encodes action space. *Neuron* **95**, 1171–1180.e1177 (2017). 28858619 10.1016/j.neuron.2017.08.015 PMC5584673

[34] Markowitz, J. E. et al. The striatum organizes 3D behavior via moment-to-moment action selection. *Cell* **174**, 44–58.e17 (2018). 29779950 10.1016/j.cell.2018.04.019 PMC6026065

[35] Weglage, M. et al. Complete representation of action space and value in all dorsal striatal pathways. *Cell Rep.* **36**, 109437 (2021). 34320355 10.1016/j.celrep.2021.109437

[36] Arber, S. & Costa, R. M. Connecting neuronal circuits for movement. *Science* **360**, 1403–1404 (2018). 29954969 10.1126/science.aat5994

[37] Klaus, A., Alves da Silva, J. & Costa, R. M. What, if, and when to move: basal ganglia circuits and self-paced action initiation. *Annu Rev. Neurosci.* **42**, 459–483 (2019). 31018098 10.1146/annurev-neuro-072116-031033

[38] Thura, D. & Cisek, P. The basal ganglia do not select reach targets but control the urgency of commitment. *Neuron* **95**, 1160–1170.e1165 (2017). 28823728 10.1016/j.neuron.2017.07.039

[39] Turner, R. S. & Anderson, M. E. Pallidal discharge related to the kinematics of reaching movements in two dimensions. *J. Neurophysiol.* **77**, 1051–1074 (1997). 10.1152/jn.1997.77.3.1051 9084582

[40] Chevalier, G., Vacher, S., Deniau, J. M. & Desban, M. Disinhibition as a basic process in the expression of striatal functions. I. The striato-nigral influence on tecto-spinal/tecto-diencephalic neurons. *Brain Res.* **334**, 215–226 (1985). 2859912 10.1016/0006-8993(85)90213-6

[41] Deniau, J. M. & Chevalier, G. Disinhibition as a basic process in the expression of striatal functions. II. The striato-nigral influence on thalamocortical cells of the ventromedial thalamic nucleus. *Brain Res.* **334**, 227–233 (1985). 3995318 10.1016/0006-8993(85)90214-8

[42] Grillner, S. & Robertson, B. The basal ganglia over 500 million years. *Curr. Biol.* **26**, R1088–R1100 (2016). 27780050 10.1016/j.cub.2016.06.041

[43] Hikosaka, O., Takikawa, Y. & Kawagoe, R. Role of the basal ganglia in the control of purposive saccadic eye movements. *Physiol. Rev.* **80**, 953–978 (2000). 10893428 10.1152/physrev.2000.80.3.953

[44] Mink, J. W. The basal ganglia: focused selection and inhibition of competing motor programs. *Prog. Neurobiol.* **50**, 381–425 (1996). 9004351 10.1016/s0301-0082(96)00042-1

[45] Redgrave, P., Vautrelle, N. & Reynolds, J. N. Functional properties of the basal ganglia’s re-entrant loop architecture: selection and reinforcement. *Neuroscience* **198**, 138–151 (2011). 21821101 10.1016/j.neuroscience.2011.07.060

[46] Friedman, A. et al. A corticostriatal path targeting striosomes controls decision-making under conflict. *Cell* **161**, 1320–1333 (2015). 26027737 10.1016/j.cell.2015.04.049 PMC4477966

[47] Terra, H. et al. Prefrontal cortical projection neurons targeting dorsomedial striatum control behavioral inhibition. *Curr. Biol.* **30**, 4188–4200.e4185 (2020). 32888489 10.1016/j.cub.2020.08.031

[48] Evans, R. C. et al. Functional dissection of basal ganglia inhibitory inputs onto substantia nigra dopaminergic neurons. *Cell Rep.* **32**, 108156 (2020). 32937133 10.1016/j.celrep.2020.108156 PMC9887718

[49] Centonze, D. et al. Unilateral dopamine denervation blocks corticostriatal LTP. *J. Neurophysiol.* **82**, 3575–3579 (1999). 10601483 10.1152/jn.1999.82.6.3575

[50] Reynolds, J. N. & Wickens, J. R. Dopamine-dependent plasticity of corticostriatal synapses. *Neural Netw.* **15**, 507–521 (2002). 12371508 10.1016/s0893-6080(02)00045-x

[51] Kreitzer, A. C. & Malenka, R. C. Striatal plasticity and basal ganglia circuit function. *Neuron* **60**, 543–554 (2008). 19038213 10.1016/j.neuron.2008.11.005 PMC2724179

[52] Montague, P. R., Dayan, P. & Sejnowski, T. J. A framework for mesencephalic dopamine systems based on predictive Hebbian learning. *J. Neurosci.* **16**, 1936–1947 (1996). 8774460 10.1523/JNEUROSCI.16-05-01936.1996 PMC6578666

[53] Nadel, J. A. et al. Optogenetic stimulation of striatal patches modifies habit formation and inhibits dopamine release. *Sci. Rep.* **11**, 19847 (2021). 34615966 10.1038/s41598-021-99350-5 PMC8494762

[54] Bernklau, T. W., Righetti, B., Mehrke, L. S. & Jacob, S. N. Striatal dopamine signals reflect perceived cue-action-outcome associations in mice. *Nat. Neurosci.* **27**, 747–757 (2024). 38291283 10.1038/s41593-023-01567-2 PMC11001585

[55] Li, J. et al. The orbitofrontal cortex to striatal cholinergic interneuron circuit controls cognitive flexibility shaping alcohol-seeking behavior. *Biol. Psychiatry* **97**, 614–626 (2025). 39396737 10.1016/j.biopsych.2024.10.005

[56] Baker, P. M. & Ragozzino, M. E. Contralateral disconnection of the rat prelimbic cortex and dorsomedial striatum impairs cue-guided behavioral switching. *Learn Mem.* **21**, 368–379 (2014). 25028395 10.1101/lm.034819.114 PMC4105715

[57] Stalnaker, T. A., Berg, B., Aujla, N. & Schoenbaum, G. Cholinergic interneurons use orbitofrontal input to track beliefs about current state. *J. Neurosci.* **36**, 6242–6257 (2016). 27277802 10.1523/JNEUROSCI.0157-16.2016 PMC4899526

[58] Kelley, R. et al. A human prefrontal-subthalamic circuit for cognitive control. *Brain* **141**, 205–216 (2018). 29190362 10.1093/brain/awx300 PMC5837669

[59] Chen, W. et al. Prefrontal-subthalamic hyperdirect pathway modulates movement inhibition in humans. *Neuron* **106**, 579–588.e573 (2020). 32155442 10.1016/j.neuron.2020.02.012 PMC7274135

[60] Yin, H. H. & Knowlton, B. J. The role of the basal ganglia in habit formation. *Nat. Rev. Neurosci.* **7**, 464–476 (2006). 16715055 10.1038/nrn1919

[61] Doyon, J. et al. Contributions of the basal ganglia and functionally related brain structures to motor learning. *Behav. Brain Res.* **199**, 61–75 (2009). 19061920 10.1016/j.bbr.2008.11.012

[62] Knowlton, B. J., Mangels, J. A. & Squire, L. R. A neostriatal habit learning system in humans. *Science* **273**, 1399–1402 (1996). 8703077 10.1126/science.273.5280.1399

[63] Saint-Cyr, J. A., Taylor, A. E. & Lang, A. E. Procedural learning and neostriatal dysfunction in man. *Brain* **111**, 941–959 (1988). 2969762 10.1093/brain/111.4.941

[64] Yin, H. H. et al. Dynamic reorganization of striatal circuits during the acquisition and consolidation of a skill. *Nat. Neurosci.* **12**, 333–341 (2009). 19198605 10.1038/nn.2261 PMC2774785

[65] Malvaez, M. & Wassum, K. M. Regulation of habit formation in the dorsal striatum. *Curr. Opin. Behav. Sci.* **20**, 67–74 (2018). 29713658 10.1016/j.cobeha.2017.11.005 PMC5920535

[66] Alloway, K. D., Smith, J. B., Mowery, T. M. & Watson, G. D. R. Sensory processing in the dorsolateral striatum: the contribution of thalamostriatal pathways. *Front. Syst. Neurosci.* **11**, 53 (2017). 28790899 10.3389/fnsys.2017.00053 PMC5524679

[67] Kato, S. et al. Action selection and flexible switching controlled by the intralaminar thalamic neurons. *Cell Rep.* **22**, 2370–2382 (2018). 29490273 10.1016/j.celrep.2018.02.016

[68] Kato, S., Nishizawa, K. & Kobayashi, K. Thalamostriatal system controls the acquisition, performance, and flexibility of learning behavior. *Front. Syst. Neurosci.* **15**, 729389 (2021). 34733142 10.3389/fnsys.2021.729389 PMC8558393

[69] Fisher, S. D. & Reynolds, J. N. The intralaminar thalamus-an expressway linking visual stimuli to circuits determining agency and action selection. *Front. Behav. Neurosci.* **8**, 115 (2014). 24765070 10.3389/fnbeh.2014.00115 PMC3980097

[70] Day-Brown, J. D., Wei, H., Chomsung, R. D., Petry, H. M. & Bickford, M. E. Pulvinar projections to the striatum and amygdala in the tree shrew. *Front. Neuroanat.* **4**, 143 (2010). 21120139 10.3389/fnana.2010.00143 PMC2991220

[71] Pidoux, M., Mahon, S., Deniau, J. M. & Charpier, S. Integration and propagation of somatosensory responses in the corticostriatal pathway: an intracellular study in vivo. *J. Physiol.* **589**, 263–281 (2011). 21059765 10.1113/jphysiol.2010.199646 PMC3043532

[72] Reig, R. & Silberberg, G. Distinct corticostriatal and intracortical pathways mediate bilateral sensory responses in the striatum. *Cereb. Cortex* **26**, 4405–4415 (2016). 27664965 10.1093/cercor/bhw268 PMC5193142

[73] Graybiel, A. M. & Grafton, S. T. The striatum: where skills and habits meet. *Cold Spring Harb. Perspect. Biol.* **7**, a021691 (2015). 26238359 10.1101/cshperspect.a021691 PMC4526748

[74] Alexander, G. E., DeLong, M. R. & Strick, P. L. Parallel organization of functionally segregated circuits linking basal ganglia and cortex. *Annu Rev. Neurosci.* **9**, 357–381 (1986). 3085570 10.1146/annurev.ne.09.030186.002041

[75] Balleine, B. W., Dezfouli, A., Ito, M. & Doya, K. Hierarchical control of goal-directed action in the cortical–basal ganglia network. *Curr. Opin. Behav. Sci.* **5**, 1–7 (2015).

[76] Haber, S. N. The primate basal ganglia: parallel and integrative networks. *J. Chem. Neuroanat.* **26**, 317–330 (2003). 14729134 10.1016/j.jchemneu.2003.10.003

[77] Korb, F. M., Jiang, J., King, J. A. & Egner, T. Hierarchically organized medial frontal cortex-basal ganglia loops selectively control task- and response-selection. *J. Neurosci.* **37**, 7893–7905 (2017). 28716966 10.1523/JNEUROSCI.3289-16.2017 PMC5559764

[78] Lee, J., Wang, W. & Sabatini, B. L. Anatomically segregated basal ganglia pathways allow parallel behavioral modulation. *Nat. Neurosci.* **23**, 1388–1398 (2020). 32989293 10.1038/s41593-020-00712-5 PMC7606600

[79] Rusu, S. I. & Pennartz, C. M. A. Learning, memory and consolidation mechanisms for behavioral control in hierarchically organized cortico-basal ganglia systems. *Hippocampus* **30**, 73–98 (2020). 31617622 10.1002/hipo.23167 PMC6972576

[80] Samejima, K. & Doya, K. Multiple representations of belief states and action values in corticobasal ganglia loops. *Ann. N.Y. Acad. Sci.* **1104**, 213–228 (2007). 17435124 10.1196/annals.1390.024

[81] Haber, S. N. & Calzavara, R. The cortico-basal ganglia integrative network: the role of the thalamus. *Brain Res. Bull.* **78**, 69–74 (2009). 18950692 10.1016/j.brainresbull.2008.09.013 PMC4459637

[82] Kuramoto, E. et al. Individual mediodorsal thalamic neurons project to multiple areas of the rat prefrontal cortex: a single neuron-tracing study using virus vectors. *J. Comp. Neurol.* **525**, 166–185 (2017). 27275581 10.1002/cne.24054

[83] Mitchell, A. S. The mediodorsal thalamus as a higher order thalamic relay nucleus important for learning and decision-making. *Neurosci. Biobehav Rev.* **54**, 76–88 (2015). 25757689 10.1016/j.neubiorev.2015.03.001

[84] Tang, H., Costa, V. D., Bartolo, R. & Averbeck, B. B. Differential coding of goals and actions in ventral and dorsal corticostriatal circuits during goal-directed behavior. *Cell Rep.* **38**, 110198 (2022). 34986350 10.1016/j.celrep.2021.110198 PMC9608360

[85] Murakami, M., Shteingart, H., Loewenstein, Y. & Mainen, Z. F. Distinct sources of deterministic and stochastic components of action timing decisions in rodent frontal cortex. *Neuron* **94**, 908–919.e907 (2017). 28521140 10.1016/j.neuron.2017.04.040

[86] Alexander, W. H. & Brown, J. W. Hierarchical error representation: a computational model of anterior cingulate and dorsolateral prefrontal cortex. *Neural Comput.* **27**, 2354–2410 (2015). 26378874 10.1162/NECO_a_00779

[87] Alexander, W. H. & Brown, J. W. Frontal cortex function as derived from hierarchical predictive coding. *Sci. Rep.* **8**, 3843 (2018). 29497060 10.1038/s41598-018-21407-9 PMC5832795

[88] Fuster, J. M. The prefrontal cortex makes the brain a preadaptive system. *Proc. IEEE* **102**, 417–426 (2014).

[89] Soltani, A. & Koechlin, E. Computational models of adaptive behavior and prefrontal cortex. *Neuropsychopharmacology* **47**, 58–71 (2022). 34389808 10.1038/s41386-021-01123-1 PMC8617006

[90] Cisek, P. Evolution of behavioural control from chordates to primates. *Philos. Trans. R. Soc. Lond. B Biol. Sci.* **377**, 20200522 (2022). 34957850 10.1098/rstb.2020.0522 PMC8710891

[91] Bargh, J. A., Gollwitzer, P. M., Lee-Chai, A., Barndollar, K. & Trotschel, R. The automated will: nonconscious activation and pursuit of behavioral goals. *J. Pers. Soc. Psychol.* **81**, 1014–1027 (2001). 11761304 PMC3005626

[92] Aarts, H. & Dijksterhuis, A. Habits as knowledge structures: automaticity in goal-directed behavior. *J. Pers. Soc. Psychol.* **78**, 53–63 (2000). 10653505 10.1037//0022-3514.78.1.53

[93] Verplanken, B. & Aarts, H. Habit, attitude, and planned behaviour: is habit an empty construct or an interesting case of goal-directed automaticity? *Eur. Rev. Soc. Psychol.* **10**, 101–134 (1999).

[94] Burton, A. C., Nakamura, K. & Roesch, M. R. From ventral-medial to dorsal-lateral striatum: neural correlates of reward-guided decision-making. *Neurobiol. Learn Mem.* **117**, 51–59 (2015). 24858182 10.1016/j.nlm.2014.05.003 PMC4240773

[95] Ito, M. & Doya, K. Multiple representations and algorithms for reinforcement learning in the cortico-basal ganglia circuit. *Curr. Opin. Neurobiol.* **21**, 368–373 (2011). 21531544 10.1016/j.conb.2011.04.001

[96] Balsters, J. H., Zerbi, V., Sallet, J., Wenderoth, N. & Mars, R. B. Primate homologs of mouse cortico-striatal circuits. *Elife* **9**, e53680 (2020). 10.7554/eLife.53680 PMC7162658 32298231

[97] Gao, L. et al. Single-neuron projectome of mouse prefrontal cortex. *Nat. Neurosci.* **25**, 515–529 (2022). 35361973 10.1038/s41593-022-01041-5

[98] Hintiryan, H. et al. The mouse cortico-striatal projectome. *Nat. Neurosci.* **19**, 1100–1114 (2016). 27322419 10.1038/nn.4332 PMC5564682

[99] Voorn, P., Vanderschuren, L. J., Groenewegen, H. J., Robbins, T. W. & Pennartz, C. M. Putting a spin on the dorsal-ventral divide of the striatum. *Trends Neurosci.* **27**, 468–474 (2004). 15271494 10.1016/j.tins.2004.06.006

[100] Ito, M. & Doya, K. Distinct neural representation in the dorsolateral, dorsomedial, and ventral parts of the striatum during fixed- and free-choice tasks. *J. Neurosci.* **35**, 3499–3514 (2015). 25716849 10.1523/JNEUROSCI.1962-14.2015 PMC4339358

[101] Heilbronner, S. R., Rodriguez-Romaguera, J., Quirk, G. J., Groenewegen, H. J. & Haber, S. N. Circuit-based corticostriatal homologies between rat and primate. *Biol. Psychiatry* **80**, 509–521 (2016). 27450032 10.1016/j.biopsych.2016.05.012 PMC5438202

[102] Mailly, P., Aliane, V., Groenewegen, H. J., Haber, S. N. & Deniau, J. M. The rat prefrontostriatal system analyzed in 3D: evidence for multiple interacting functional units. *J. Neurosci.* **33**, 5718–5727 (2013). 23536085 10.1523/JNEUROSCI.5248-12.2013 PMC6705071

[103] McGinty, V. B., Lardeux, S., Taha, S. A., Kim, J. J. & Nicola, S. M. Invigoration of reward seeking by cue and proximity encoding in the nucleus accumbens. *Neuron* **78**, 910–922 (2013). 23764290 10.1016/j.neuron.2013.04.010 PMC3954588

[104] Salamone, J. D. & Correa, M. The neurobiology of activational aspects of motivation: exertion of effort, effort-based decision making, and the role of dopamine. *Annu Rev. Psychol.* **75**, 1–32 (2024). 37788571 10.1146/annurev-psych-020223-012208

[105] Soares-Cunha, C. et al. Activation of D2 dopamine receptor-expressing neurons in the nucleus accumbens increases motivation. *Nat. Commun.* **7**, 11829 (2016). 27337658 10.1038/ncomms11829 PMC4931006

[106] Olds, J. & Milner, P. Positive reinforcement produced by electrical stimulation of septal area and other regions of rat brain. *J. Comp. Physiol. Psychol.* **47**, 419–427 (1954). 13233369 10.1037/h0058775

[107] Heath, R. G. Pleasure and brain activity in man. Deep and surface electroencephalograms during orgasm. *J. Nerv. Ment. Dis.* **154**, 3–18 (1972). 5007439 10.1097/00005053-197201000-00002

[108] Pecina, S. & Berridge, K. C. Hedonic hot spot in nucleus accumbens shell: Where do mu-opioids cause increased hedonic impact of sweetness? *J. Neurosci.* **25**, 11777–11786 (2005). 16354936 10.1523/JNEUROSCI.2329-05.2005 PMC6726018

[109] Schlaepfer, T. E. et al. Deep brain stimulation to reward circuitry alleviates anhedonia in refractory major depression. *Neuropsychopharmacology* **33**, 368–377 (2008). 17429407 10.1038/sj.npp.1301408

[110] Smith, K. S., Berridge, K. C. & Aldridge, J. W. Disentangling pleasure from incentive salience and learning signals in brain reward circuitry. *Proc. Natl. Acad. Sci. USA* **108**, E255–E264 (2011). 21670308 10.1073/pnas.1101920108 PMC3131314

[111] Berridge, K. C. & Kringelbach, M. L. Pleasure systems in the brain. *Neuron* **86**, 646–664 (2015). 25950633 10.1016/j.neuron.2015.02.018 PMC4425246

[112] Cole, S. L., Robinson, M. J. F. & Berridge, K. C. Optogenetic self-stimulation in the nucleus accumbens: D1 reward versus D2 ambivalence. *PLoS ONE* **13**, e0207694 (2018). 30496206 10.1371/journal.pone.0207694 PMC6264872

[113] du Hoffmann, J. & Nicola, S. M. Dopamine invigorates reward seeking by promoting cue-evoked excitation in the nucleus accumbens. *J. Neurosci.* **34**, 14349–14364 (2014). 25339748 10.1523/JNEUROSCI.3492-14.2014 PMC4205557

[114] Nicola, S. M. The flexible approach hypothesis: unification of effort and cue-responding hypotheses for the role of nucleus accumbens dopamine in the activation of reward-seeking behavior. *J. Neurosci.* **30**, 16585–16600 (2010). 21147998 10.1523/JNEUROSCI.3958-10.2010 PMC3030450

[115] Schultz, W., Apicella, P., Scarnati, E. & Ljungberg, T. Neuronal activity in monkey ventral striatum related to the expectation of reward. *J. Neurosci.* **12**, 4595–4610 (1992). 1464759 10.1523/JNEUROSCI.12-12-04595.1992 PMC6575755

[116] Setlow, B., Schoenbaum, G. & Gallagher, M. Neural encoding in ventral striatum during olfactory discrimination learning. *Neuron* **38**, 625–636 (2003). 12765613 10.1016/s0896-6273(03)00264-2

[117] Strait, C. E., Sleezer, B. J. & Hayden, B. Y. Signatures of value comparison in ventral striatum neurons. *PLoS Biol.* **13**, e1002173 (2015). 26086735 10.1371/journal.pbio.1002173 PMC4472856

[118] Roesch, M. R., Singh, T., Brown, P. L., Mullins, S. E. & Schoenbaum, G. Ventral striatal neurons encode the value of the chosen action in rats deciding between differently delayed or sized rewards. *J. Neurosci.* **29**, 13365–13376 (2009). 19846724 10.1523/JNEUROSCI.2572-09.2009 PMC2788608

[119] Vega-Villar, M., Horvitz, J. C. & Nicola, S. M. NMDA receptor-dependent plasticity in the nucleus accumbens connects reward-predictive cues to approach responses. *Nat. Commun.* **10**, 4429 (2019). 31562332 10.1038/s41467-019-12387-z PMC6764993

[120] Di Chiara, G. & Imperato, A. Drugs abused by humans preferentially increase synaptic dopamine concentrations in the mesolimbic system of freely moving rats. *Proc. Natl. Acad. Sci. USA* **85**, 5274–5278 (1988). 2899326 10.1073/pnas.85.14.5274 PMC281732

[121] Luscher, C., Robbins, T. W. & Everitt, B. J. The transition to compulsion in addiction. *Nat. Rev. Neurosci.* **21**, 247–263 (2020). 32231315 10.1038/s41583-020-0289-z PMC7610550

[122] Deniau, J. M., Menetrey, A. & Thierry, A. M. Indirect nucleus accumbens input to the prefrontal cortex via the substantia nigra pars reticulata: a combined anatomical and electrophysiological study in the rat. *Neuroscience* **61**, 533–545 (1994). 7526269 10.1016/0306-4522(94)90432-4

[123] Haber, S. N., Lynd, E., Klein, C. & Groenewegen, H. J. Topographic organization of the ventral striatal efferent projections in the rhesus monkey: an anterograde tracing study. *J. Comp. Neurol.* **293**, 282–298 (1990). 19189717 10.1002/cne.902930210

[124] Watabe-Uchida, M., Zhu, L., Ogawa, S. K., Vamanrao, A. & Uchida, N. Whole-brain mapping of direct inputs to midbrain dopamine neurons. *Neuron* **74**, 858–873 (2012). 22681690 10.1016/j.neuron.2012.03.017

[125] Ragozzino, M. E. The contribution of the medial prefrontal cortex, orbitofrontal cortex, and dorsomedial striatum to behavioral flexibility. *Ann. N.Y. Acad. Sci.* **1121**, 355–375 (2007). 17698989 10.1196/annals.1401.013

[126] Hollerman, J. R., Tremblay, L. & Schultz, W. Influence of reward expectation on behavior-related neuronal activity in primate striatum. *J. Neurophysiol.* **80**, 947–963 (1998). 9705481 10.1152/jn.1998.80.2.947

[127] Oyama, K. et al. Discrete coding of stimulus value, reward expectation, and reward prediction error in the dorsal striatum. *J. Neurophysiol.* **114**, 2600–2615 (2015). 26378201 10.1152/jn.00097.2015 PMC4637368

[128] Choi, K. et al. Distributed processing for value-based choice by prelimbic circuits targeting anterior-posterior dorsal striatal subregions in male mice. *Nat. Commun.* **14**, 1920 (2023). 37024449 10.1038/s41467-023-36795-4 PMC10079960

[129] Jin, X., Tecuapetla, F. & Costa, R. M. Basal ganglia subcircuits distinctively encode the parsing and concatenation of action sequences. *Nat. Neurosci.* **17**, 423–430 (2014). 24464039 10.1038/nn.3632 PMC3955116

[130] Minkowicz, S. et al. Striatal ensemble activity in an innate naturalistic behavior. *bioRxiv*. 10.1101/2023.02.23.529669 (2023).

[131] Markowitz, J. E. A groom with a view. *Elife* **12**, e88595 (2023). 10.7554/eLife.88595 PMC10188107 37191296

[132] Jin, X. & Costa, R. M. Start/stop signals emerge in nigrostriatal circuits during sequence learning. *Nature* **466**, 457–462 (2010). 20651684 10.1038/nature09263 PMC3477867

[133] Butcher, L. L. & Hodge, G. K. Postnatal development of acetylcholinesterase in the caudate-putamen nucleus and substantia nigra of rats. *Brain Res.* **106**, 223–240 (1976). 1276870 10.1016/0006-8993(76)91022-2

[134] Graybiel, A. M., Ragsdale, C. W. Jr., Yoneoka, E. S. & Elde, R. P. An immunohistochemical study of enkephalins and other neuropeptides in the striatum of the cat with evidence that the opiate peptides are arranged to form mosaic patterns in register with the striosomal compartments visible by acetylcholinesterase staining. *Neuroscience* **6**, 377–397 (1981). 6164013 10.1016/0306-4522(81)90131-7

[135] Miyamoto, Y., Katayama, S., Shigematsu, N., Nishi, A. & Fukuda, T. Striosome-based map of the mouse striatum that is conformable to both cortical afferent topography and uneven distributions of dopamine D1 and D2 receptor-expressing cells. *Brain Struct. Funct.* **223**, 4275–4291 (2018). 30203304 10.1007/s00429-018-1749-3 PMC6267261

[136] McGregor, M. M. et al. Functionally distinct connectivity of developmentally targeted striosome neurons. *Cell Rep.* **29**, 1419–1428.e1415 (2019). 31693884 10.1016/j.celrep.2019.09.076 PMC6866662

[137] Bolam, J. P., Izzo, P. N. & Graybiel, A. M. Cellular substrate of the histochemically defined striosome/matrix system of the caudate nucleus: a combined Golgi and immunocytochemical study in cat and ferret. *Neuroscience* **24**, 853–875 (1988). 2454418 10.1016/0306-4522(88)90073-5

[138] Lopez-Huerta, V. G. et al. The neostriatum: two entities, one structure? *Brain Struct. Funct.* **221**, 1737–1749 (2016). 25652680 10.1007/s00429-015-1000-4 PMC4819794

[139] Bloem, B. et al. Multiplexed action-outcome representation by striatal striosome-matrix compartments detected with a mouse cost-benefit foraging task. *Nat. Commun.* **13**, 1541 (2022). 35318343 10.1038/s41467-022-28983-5 PMC8941061

[140] Friedman, A. et al. Striosomes mediate value-based learning vulnerable in age and a Huntington’s disease model. *Cell* **183**, 918–934.e949 (2020). 33113354 10.1016/j.cell.2020.09.060 PMC7932131

[141] Yoshizawa, T., Ito, M. & Doya, K. Reward-predictive neural activities in striatal striosome compartments. *eNeuro* **5**, ENEURO.0367-17 (2018). 10.1523/ENEURO.0367-17.2018 PMC5804148 29430520

[142] Houk, J. C., Adams, J. L. & Barto, A. G. in *Models of Information Processing in the Basal Ganglia* (The MIT Press, 1994).

[143] Suri, R. E. & Schultz, W. A neural network model with dopamine-like reinforcement signal that learns a spatial delayed response task. *Neuroscience* **91**, 871–890 (1999). 10391468 10.1016/s0306-4522(98)00697-6

[144] Bhatnagar, S., Sutton, R. S., Ghavamzadeh, M. & Lee, M. Natural actor-critic algorithms. *Automatica* **45**, 2471–2482 (2009).

[145] Joel, D., Niv, Y. & Ruppin, E. Actor-critic models of the basal ganglia: new anatomical and computational perspectives. *Neural Netw.* **15**, 535–547 (2002). 12371510 10.1016/s0893-6080(02)00047-3

[146] Suri, R. E., Bargas, J. & Arbib, M. A. Modeling functions of striatal dopamine modulation in learning and planning. *Neuroscience* **103**, 65–85 (2001). 11311788 10.1016/s0306-4522(00)00554-6

[147] Daw, N. D., Gershman, S. J., Seymour, B., Dayan, P. & Dolan, R. J. Model-based influences on humans’ choices and striatal prediction errors. *Neuron* **69**, 1204–1215 (2011). 21435563 10.1016/j.neuron.2011.02.027 PMC3077926

[148] Daw, N. D., Niv, Y. & Dayan, P. Uncertainty-based competition between prefrontal and dorsolateral striatal systems for behavioral control. *Nat. Neurosci.* **8**, 1704–1711 (2005). 16286932 10.1038/nn1560

[149] Doya, K. What are the computations of the cerebellum, the basal ganglia and the cerebral cortex? *Neural Netw.* **12**, 961–974 (1999). 12662639 10.1016/s0893-6080(99)00046-5

[150] Doya, K. Complementary roles of basal ganglia and cerebellum in learning and motor control. *Curr. Opin. Neurobiol.* **10**, 732–739 (2000). 11240282 10.1016/s0959-4388(00)00153-7

[151] Crittenden, J. R. & Graybiel, A. M. Basal Ganglia disorders associated with imbalances in the striatal striosome and matrix compartments. *Front Neuroanat.* **5**, 59 (2011). 21941467 10.3389/fnana.2011.00059 PMC3171104

[152] Graybiel, A. M. & Matsushima, A. Striosomes and Matrisomes: Scaffolds for Dynamic Coupling of Volition and Action. *Annu Rev. Neurosci.* **46**, 359–380 (2023). 37068787 10.1146/annurev-neuro-121522-025740

[153] Atallah, H. E., Lopez-Paniagua, D., Rudy, J. W. & O’Reilly, R. C. Separate neural substrates for skill learning and performance in the ventral and dorsal striatum. *Nat. Neurosci.* **10**, 126–131 (2007). 17187065 10.1038/nn1817

[154] Bornstein, A. M. & Daw, N. D. Multiplicity of control in the basal ganglia: computational roles of striatal subregions. *Curr. Opin. Neurobiol.* **21**, 374–380 (2011). 21429734 10.1016/j.conb.2011.02.009 PMC3269306

[155] O’Doherty, J. et al. Dissociable roles of ventral and dorsal striatum in instrumental conditioning. *Science* **304**, 452–454 (2004). 15087550 10.1126/science.1094285

[156] Jongen-Relo, A. L., Groenewegen, H. J. & Voorn, P. Evidence for a multi-compartmental histochemical organization of the nucleus accumbens in the rat. *J. Comp. Neurol.* **337**, 267–276 (1993). 8277000 10.1002/cne.903370207

[157] Mansour, A. et al. Mu, delta, and kappa opioid receptor mRNA expression in the rat CNS: an in situ hybridization study. *J. Comp. Neurol.* **350**, 412–438 (1994). 7884049 10.1002/cne.903500307

[158] Szydlowski, S. N. et al. Target selectivity of feedforward inhibition by striatal fast-spiking interneurons. *J. Neurosci.* **33**, 1678–1683 (2013). 23345240 10.1523/JNEUROSCI.3572-12.2013 PMC6618742

[159] Tepper, J. M., Wilson, C. J. & Koos, T. Feedforward and feedback inhibition in neostriatal GABAergic spiny neurons. *Brain Res. Rev.* **58**, 272–281 (2008). 18054796 10.1016/j.brainresrev.2007.10.008 PMC2562631

[160] Czubayko, U. & Plenz, D. Fast synaptic transmission between striatal spiny projection neurons. *Proc. Natl. Acad. Sci. USA* **99**, 15764–15769 (2002). 12438690 10.1073/pnas.242428599 PMC137790

[161] Taverna, S., Ilijic, E. & Surmeier, D. J. Recurrent collateral connections of striatal medium spiny neurons are disrupted in models of Parkinson’s disease. *J. Neurosci.* **28**, 5504–5512 (2008). 18495884 10.1523/JNEUROSCI.5493-07.2008 PMC3235738

[162] Gurney, K., Prescott, T. J. & Redgrave, P. A computational model of action selection in the basal ganglia. I. A new functional anatomy. *Biol. Cyber.* **84**, 401–410 (2001). 10.1007/PL00007984 11417052

[163] Doig, N. M., Moss, J. & Bolam, J. P. Cortical and thalamic innervation of direct and indirect pathway medium-sized spiny neurons in mouse striatum. *J. Neurosci.* **30**, 14610–14618 (2010). 21048118 10.1523/JNEUROSCI.1623-10.2010 PMC6633626

[164] Hunnicutt, B. J. et al. A comprehensive excitatory input map of the striatum reveals novel functional organization. *Elife* **5**, e19103 (2016). 10.7554/eLife.19103 PMC5207773 27892854

[165] Calabresi, P. et al. Synaptic transmission in the striatum: from plasticity to neurodegeneration. *Prog. Neurobiol.* **61**, 231–265 (2000). 10727775 10.1016/s0301-0082(99)00030-1

[166] Martiros, N., Kapoor, V., Kim, S. E. & Murthy, V. N. Distinct representation of cue-outcome association by D1 and D2 neurons in the ventral striatum’s olfactory tubercle. *Elife* **11**, e75463 (2022). 10.7554/eLife.75463 PMC9203051 35708179

[167] Rothenhoefer, K. M. et al. Effects of ventral striatum lesions on stimulus-based versus action-based reinforcement learning. *J. Neurosci.* **37**, 6902–6914 (2017). 28626011 10.1523/JNEUROSCI.0631-17.2017 PMC5518420

[168] Hassani, O. K., Cromwell, H. C. & Schultz, W. Influence of expectation of different rewards on behavior-related neuronal activity in the striatum. *J. Neurophysiol.* **85**, 2477–2489 (2001). 11387394 10.1152/jn.2001.85.6.2477

[169] Atallah, H. E., McCool, A. D., Howe, M. W. & Graybiel, A. M. Neurons in the ventral striatum exhibit cell-type-specific representations of outcome during learning. *Neuron* **82**, 1145–1156 (2014). 24908491 10.1016/j.neuron.2014.04.021 PMC4108162

[170] Domingues, A. V. et al. Dynamic representation of appetitive and aversive stimuli in nucleus accumbens shell D1- and D2-medium spiny neurons. *Nat. Commun.* **16**, 59 (2025). 39746997 10.1038/s41467-024-55269-9 PMC11696804

[171] Roitman, M. F., Wheeler, R. A. & Carelli, R. M. Nucleus accumbens neurons are innately tuned for rewarding and aversive taste stimuli, encode their predictors, and are linked to motor output. *Neuron* **45**, 587–597 (2005). 15721244 10.1016/j.neuron.2004.12.055

[172] Cerri, D. H., Saddoris, M. P. & Carelli, R. M. Nucleus accumbens core neurons encode value-independent associations necessary for sensory preconditioning. *Behav. Neurosci.* **128**, 567–578 (2014). 25244086 10.1037/a0037797 PMC4176687

[173] Moss, M. M., Zatka-Haas, P., Harris, K. D., Carandini, M. & Lak, A. Dopamine axons in dorsal striatum encode contralateral visual stimuli and choices. *J. Neurosci.* **41**, 7197–7205 (2021). 34253628 10.1523/JNEUROSCI.0490-21.2021 PMC8387116

[174] Peters, A. J., Fabre, J. M. J., Steinmetz, N. A., Harris, K. D. & Carandini, M. Striatal activity topographically reflects cortical activity. *Nature* **591**, 420–425 (2021). 33473213 10.1038/s41586-020-03166-8 PMC7612253

[175] Wang, L., Rangarajan, K. V., Gerfen, C. R. & Krauzlis, R. J. Activation of striatal neurons causes a perceptual decision bias during visual change detection in mice. *Neuron* **97**, 1369–1381.e1365 (2018). 29503185 10.1016/j.neuron.2018.01.049 PMC5866220

[176] Fuster, J. M. The prefrontal cortex–an update: time is of the essence. *Neuron* **30**, 319–333 (2001). 11394996 10.1016/s0896-6273(01)00285-9

[177] Fuster, J. M. Behavioral electrophysiology of the prefrontal cortex. *Trends Neurosci.* **7**, 408–414 (1984).

[178] Carter, C. S. et al. Anterior cingulate cortex, error detection, and the online monitoring of performance. *Science* **280**, 747–749 (1998). 9563953 10.1126/science.280.5364.747

[179] Miller, E. K. & Cohen, J. D. An integrative theory of prefrontal cortex function. *Annu Rev. Neurosci.* **24**, 167–202 (2001). 11283309 10.1146/annurev.neuro.24.1.167

[180] Shenhav, A., Botvinick, M. M. & Cohen, J. D. The expected value of control: an integrative theory of anterior cingulate cortex function. *Neuron* **79**, 217–240 (2013). 23889930 10.1016/j.neuron.2013.07.007 PMC3767969

[181] Passingham, R. E. & Wise, S. P. *The Neurobiology of the Prefrontal Cortex: Anatomy, Evolution, and the Origin of Insight* 1st edn, (Oxford Univ. Press, 2012).

[182] Schoenbaum, G., Chiba, A. A. & Gallagher, M. Orbitofrontal cortex and basolateral amygdala encode expected outcomes during learning. *Nat. Neurosci.* **1**, 155–159 (1998). 10195132 10.1038/407

[183] Schoenbaum, G., Setlow, B., Saddoris, M. P. & Gallagher, M. Encoding predicted outcome and acquired value in orbitofrontal cortex during cue sampling depends upon input from basolateral amygdala. *Neuron* **39**, 855–867 (2003). 12948451 10.1016/s0896-6273(03)00474-4

[184] Lichtenberg, N. T. et al. Basolateral amygdala to orbitofrontal cortex projections enable cue-triggered reward expectations. *J. Neurosci.* **37**, 8374–8384 (2017). 28743727 10.1523/JNEUROSCI.0486-17.2017 PMC5577854

[185] Wilson, R. C., Takahashi, Y. K., Schoenbaum, G. & Niv, Y. Orbitofrontal cortex as a cognitive map of task space. *Neuron* **81**, 267–279 (2014). 24462094 10.1016/j.neuron.2013.11.005 PMC4001869

[186] Antzoulatos, E. G. & Miller, E. K. Differences between neural activity in prefrontal cortex and striatum during learning of novel abstract categories. *Neuron* **71**, 243–249 (2011). 21791284 10.1016/j.neuron.2011.05.040 PMC3253019

[187] Riceberg, J. S. & Shapiro, M. L. Orbitofrontal cortex signals expected outcomes with predictive codes when stable contingencies promote the integration of reward history. *J. Neurosci.* **37**, 2010–2021 (2017). 28115481 10.1523/JNEUROSCI.2951-16.2016 PMC5338752

[188] Diehl, G. W. & Redish, A. D. Differential processing of decision information in subregions of rodent medial prefrontal cortex. *Elife* **12**10.7554/eLife.82833 (2023). 10.7554/eLife.82833 PMC9848391 36652289

[189] Majumdar, A. et al. Distinct representations of economic variables across regions and projections of the frontal cortex. *Neuron* **113**, 4232–4244.e4238 (2025). 41106378 10.1016/j.neuron.2025.09.027

[190] Sul, J. H., Kim, H., Huh, N., Lee, D. & Jung, M. W. Distinct roles of rodent orbitofrontal and medial prefrontal cortex in decision making. *Neuron* **66**, 449–460 (2010). 20471357 10.1016/j.neuron.2010.03.033 PMC2872629

[191] Halassa, M. M. & Sherman, S. M. Thalamocortical Circuit Motifs: A General Framework. *Neuron* **103**, 762–770 (2019). 31487527 10.1016/j.neuron.2019.06.005 PMC6886702

[192] Sherman, S. M. & Usrey, W. M. Transthalamic pathways for cortical function. *J. Neurosci.* **44**, e0909242024 (2024). 10.1523/JNEUROSCI.0909-24.2024 PMC11358609 39197951

[193] Halassa, M. M. Fronto-thalamic architectures for cognitive algorithms. *Neuron* **98**, 237–239 (2018). 29673474 10.1016/j.neuron.2018.04.006

[194] Goldman-Rakic, P. S. Cellular basis of working memory. *Neuron* **14**, 477–485 (1995). 7695894 10.1016/0896-6273(95)90304-6

[195] Rigotti, M. et al. The importance of mixed selectivity in complex cognitive tasks. *Nature* **497**, 585–590 (2013). 23685452 10.1038/nature12160 PMC4412347

[196] Rigotti, M., Ben Dayan Rubin, D., Wang, X. J. & Fusi, S. Internal representation of task rules by recurrent dynamics: the importance of the diversity of neural responses. *Front Comput Neurosci.* **4**, 24 (2010). 21048899 10.3389/fncom.2010.00024 PMC2967380

[197] Lam, N. H. et al. Prefrontal transthalamic uncertainty processing drives flexible switching. *Nature* **637**, 127–136 (2025). 39537928 10.1038/s41586-024-08180-8 PMC11841214

[198] Mukherjee, A., Lam, N. H., Wimmer, R. D. & Halassa, M. M. Thalamic circuits for independent control of prefrontal signal and noise. *Nature* **600**, 100–104 (2021). 34614503 10.1038/s41586-021-04056-3 PMC8636261

[199] Rikhye, R. V., Gilra, A. & Halassa, M. M. Thalamic regulation of switching between cortical representations enables cognitive flexibility. *Nat. Neurosci.* **21**, 1753–1763 (2018). 30455456 10.1038/s41593-018-0269-z PMC7225728

[200] Scott, D. N., Mukherjee, A., Nassar, M. R. & Halassa, M. M. Thalamocortical architectures for flexible cognition and efficient learning. *Trends Cogn. Sci.* **28**, 739–756 (2024). 38886139 10.1016/j.tics.2024.05.006 PMC11305962

[201] Zhang, X., Mukherjee, A., Halassa, M. M. & Chen, Z. S. Mediodorsal thalamus regulates task uncertainty to enable cognitive flexibility. *Nat. Commun.* **16**, 2640 (2025). 40097445 10.1038/s41467-025-58011-1 PMC11914509

[202] Phillips, J. M. et al. Primate thalamic nuclei select abstract rules and shape prefrontal dynamics. *Neuron* **113**, 2014–2027.e2012 (2025). 40233749 10.1016/j.neuron.2025.03.021 PMC12181055

[203] Singer, W. Neuronal synchrony: a versatile code for the definition of relations? *Neuron* **24**, 49–65 (1999). 10677026 10.1016/s0896-6273(00)80821-1

[204] Buzsaki, G. & Draguhn, A. Neuronal oscillations in cortical networks. *Science* **304**, 1926–1929 (2004). 15218136 10.1126/science.1099745

[205] Fries, P. Rhythms for Cognition: Communication through Coherence. *Neuron* **88**, 220–235 (2015). 26447583 10.1016/j.neuron.2015.09.034 PMC4605134

[206] Fiebelkorn, I. C., Pinsk, M. A. & Kastner, S. The mediodorsal pulvinar coordinates the macaque fronto-parietal network during rhythmic spatial attention. *Nat. Commun.* **10**, 215 (2019). 30644391 10.1038/s41467-018-08151-4 PMC6333835

[207] Halassa, M. M. & Kastner, S. Thalamic functions in distributed cognitive control. *Nat. Neurosci.* **20**, 1669–1679 (2017). 29184210 10.1038/s41593-017-0020-1

[208] Saalmann, Y. B., Pinsk, M. A., Wang, L., Li, X. & Kastner, S. The pulvinar regulates information transmission between cortical areas based on attention demands. *Science* **337**, 753–756 (2012). 22879517 10.1126/science.1223082 PMC3714098

[209] Llinas, R., Ribary, U., Contreras, D. & Pedroarena, C. The neuronal basis for consciousness. *Philos. Trans. R. Soc. Lond. B Biol. Sci.* **353**, 1841–1849 (1998). 9854256 10.1098/rstb.1998.0336 PMC1692417

[210] Jones, E. G. The thalamic matrix and thalamocortical synchrony. *Trends Neurosci.* **24**, 595–601 (2001). 11576674 10.1016/s0166-2236(00)01922-6

[211] Tang, H., Bartolo, R. & Averbeck, B. B. Ventral frontostriatal circuitry mediates the computation of reinforcement from symbolic gains and losses. *Neuron* **112**, 3782–3795.e3785 (2024). 39321792 10.1016/j.neuron.2024.08.018 PMC11581918

[212] Morita, K., Jitsev, J. & Morrison, A. Corticostriatal circuit mechanisms of value-based action selection: Implementation of reinforcement learning algorithms and beyond. *Behav. Brain Res.* **311**, 110–121 (2016). 27173430 10.1016/j.bbr.2016.05.017

[213] Holton, E. et al. Goal commitment is supported by vmPFC through selective attention. *Nat. Hum. Behav.* **8**, 1351–1365 (2024). 38632389 10.1038/s41562-024-01844-5 PMC11272579

[214] Murray, E. A. & Rudebeck, P. H. Specializations for reward-guided decision-making in the primate ventral prefrontal cortex. *Nat. Rev. Neurosci.* **19**, 404–417 (2018). 29795133 10.1038/s41583-018-0013-4 PMC6432632

[215] Padoa-Schioppa, C. & Assad, J. A. Neurons in the orbitofrontal cortex encode economic value. *Nature* **441**, 223–226 (2006). 16633341 10.1038/nature04676 PMC2630027

[216] Padoa-Schioppa, C. & Conen, K. E. Orbitofrontal cortex: a neural circuit for economic decisions. *Neuron* **96**, 736–754 (2017). 29144973 10.1016/j.neuron.2017.09.031 PMC5726577

[217] Roy, M., Shohamy, D. & Wager, T. D. Ventromedial prefrontal-subcortical systems and the generation of affective meaning. *Trends Cogn. Sci.* **16**, 147–156 (2012). 22310704 10.1016/j.tics.2012.01.005 PMC3318966

[218] Sleezer, B. J., Castagno, M. D. & Hayden, B. Y. Rule encoding in orbitofrontal cortex and striatum guides selection. *J. Neurosci.* **36**, 11223–11237 (2016). 27807165 10.1523/JNEUROSCI.1766-16.2016 PMC5148240

[219] Rolls, E. T., Critchley, H. D., Mason, R. & Wakeman, E. A. Orbitofrontal cortex neurons: role in olfactory and visual association learning. *J. Neurophysiol.* **75**, 1970–1981 (1996). 8734596 10.1152/jn.1996.75.5.1970

[220] Alexander, W. H. & Brown, J. W. Medial prefrontal cortex as an action-outcome predictor. *Nat. Neurosci.* **14**, 1338–1344 (2011). 21926982 10.1038/nn.2921 PMC3183374

[221] Passecker, J. et al. Activity of prefrontal neurons predict future choices during gambling. *Neuron* **101**, 152–164.e157 (2019). 30528555 10.1016/j.neuron.2018.10.050 PMC6318061

[222] Bari, B. A. et al. Stable representations of decision variables for flexible behavior. *Neuron* **103**, 922–933.e927 (2019). 31280924 10.1016/j.neuron.2019.06.001 PMC7169950

[223] Akam, T. et al. The anterior cingulate cortex predicts future states to mediate model-based action selection. *Neuron* **109**, 149–163.e147 (2021). 33152266 10.1016/j.neuron.2020.10.013 PMC7837117

[224] Marquis, J. P., Killcross, S. & Haddon, J. E. Inactivation of the prelimbic, but not infralimbic, prefrontal cortex impairs the contextual control of response conflict in rats. *Eur. J. Neurosci.* **25**, 559–566 (2007). 17284198 10.1111/j.1460-9568.2006.05295.x

[225] Lak, A. et al. Orbitofrontal cortex is required for optimal waiting based on decision confidence. *Neuron* **84**, 190–201 (2014). 25242219 10.1016/j.neuron.2014.08.039 PMC4364549

[226] Erlich, J. C., Bialek, M. & Brody, C. D. A cortical substrate for memory-guided orienting in the rat. *Neuron* **72**, 330–343 (2011). 22017991 10.1016/j.neuron.2011.07.010 PMC3212026

[227] Olson, J. M., Li, J. K., Montgomery, S. E. & Nitz, D. A. Secondary motor cortex transforms spatial information into planned action during navigation. *Curr. Biol.* **30**, 1845–1854.e1844 (2020). 32302586 10.1016/j.cub.2020.03.016

[228] Liu, J. et al. The secondary motor cortex-striatum circuit contributes to suppressing inappropriate responses in perceptual decision behavior. *Neurosci. Bull.* **39**, 1544–1560 (2023). 37253985 10.1007/s12264-023-01073-2 PMC10533474

[229] Brockett, A. T., Tennyson, S. S., deBettencourt, C. A., Gaye, F. & Roesch, M. R. Anterior cingulate cortex is necessary for adaptation of action plans. *Proc. Natl. Acad. Sci. USA* **117**, 6196–6204 (2020). 32132213 10.1073/pnas.1919303117 PMC7084129

[230] Peng, Y. et al. Brain-wide population activity during reaching integrates action-mediated goal expectation. *bioRxiv*. 10.1101/2024.11.04.621878 (2024).

[231] Ragsdale, C. W. Jr. & Graybiel, A. M. The fronto-striatal projection in the cat and monkey and its relationship to inhomogeneities established by acetylcholinesterase histochemistry. *Brain Res.* **208**, 259–266 (1981). 6163506 10.1016/0006-8993(81)90556-4

[232] Donoghue, J. P. & Herkenham, M. Neostriatal projections from individual cortical fields conform to histochemically distinct striatal compartments in the rat. *Brain Res.* **365**, 397–403 (1986). 3004664 10.1016/0006-8993(86)91658-6

[233] Eblen, F. & Graybiel, A. M. Highly restricted origin of prefrontal cortical inputs to striosomes in the macaque monkey. *J. Neurosci.* **15**, 5999–6013 (1995). 7666184 10.1523/JNEUROSCI.15-09-05999.1995 PMC6577677

[234] Waugh, J. L. et al. An MRI method for parcellating the human striatum into matrix and striosome compartments in vivo. *Neuroimage* **246**, 118714 (2022). 34800665 10.1016/j.neuroimage.2021.118714 PMC9142299

[235] Ragsdale, C. W. Jr. & Graybiel, A. M. A simple ordering of neocortical areas established by the compartmental organization of their striatal projections. *Proc. Natl. Acad. Sci. USA* **87**, 6196–6199 (1990). 1696719 10.1073/pnas.87.16.6196 PMC54499

[236] Frank, M. J. & Badre, D. Mechanisms of hierarchical reinforcement learning in corticostriatal circuits 1: computational analysis. *Cereb. Cortex* **22**, 509–526 (2012). 21693490 10.1093/cercor/bhr114 PMC3278315

[237] Otto, A. R., Raio, C. M., Chiang, A., Phelps, E. A. & Daw, N. D. Working-memory capacity protects model-based learning from stress. *Proc. Natl. Acad. Sci. USA* **110**, 20941–20946 (2013). 24324166 10.1073/pnas.1312011110 PMC3876216

[238] Zuo, Z., Yang, L. Z., Wang, H. & Li, H. Working memory guides action valuation in model-based decision-making strategy. *J. Cogn. Neurosci.* **37**, 86–96 (2025). 39136553 10.1162/jocn_a_02237

[239] Neal, D. T., Wood, W. & Drolet, A. How do people adhere to goals when willpower is low? The profits (and pitfalls) of strong habits. *J. Pers. Soc. Psychol.* **104**, 959–975 (2013). 23730907 10.1037/a0032626

[240] Schwabe, L. & Wolf, O. T. Stress-induced modulation of instrumental behavior: from goal-directed to habitual control of action. *Behav. Brain Res.* **219**, 321–328 (2011). 21219935 10.1016/j.bbr.2010.12.038

[241] Haber, S. N., Fudge, J. L. & McFarland, N. R. Striatonigrostriatal pathways in primates form an ascending spiral from the shell to the dorsolateral striatum. *J. Neurosci.* **20**, 2369–2382 (2000). 10704511 10.1523/JNEUROSCI.20-06-02369.2000 PMC6772499

[242] Ikemoto, S. Dopamine reward circuitry: two projection systems from the ventral midbrain to the nucleus accumbens-olfactory tubercle complex. *Brain Res. Rev.* **56**, 27–78 (2007). 17574681 10.1016/j.brainresrev.2007.05.004 PMC2134972

[243] Nauta, W. J., Smith, G. P., Faull, R. L. & Domesick, V. B. Efferent connections and nigral afferents of the nucleus accumbens septi in the rat. *Neuroscience* **3**, 385–401 (1978). 683502 10.1016/0306-4522(78)90041-6

[244] Dong, J. et al. Molecularly distinct striatonigral neuron subtypes differentially regulate locomotion. *Nat. Commun.* **16**, 2710 (2025). 40108161 10.1038/s41467-025-58007-x PMC11923167

[245] Okunomiya, T. et al. Striosome circuitry stimulation inhibits striatal dopamine release and locomotion. *J. Neurosci.* **45**, e0457242024 (2025). 10.1523/JNEUROSCI.0457-24.2024 PMC11756628 39622644

[246] Stephenson-Jones, M., Kardamakis, A. A., Robertson, B. & Grillner, S. Independent circuits in the basal ganglia for the evaluation and selection of actions. *Proc. Natl. Acad. Sci. USA* **110**, E3670–E3679 (2013). 24003130 10.1073/pnas.1314815110 PMC3780871

[247] Stephenson-Jones, M. et al. A basal ganglia circuit for evaluating action outcomes. *Nature* **539**, 289–293 (2016). 27652894 10.1038/nature19845 PMC5161609

[248] Lazaridis, I. et al. Striosomes control dopamine via dual pathways paralleling canonical basal ganglia circuits. *Curr. Biol.* **34**, 5263–5283.e5268 (2024). 39447573 10.1016/j.cub.2024.09.070 PMC11702862

[249] Calabresi, P. et al. Dopamine and cAMP-regulated phosphoprotein 32 kDa controls both striatal long-term depression and long-term potentiation, opposing forms of synaptic plasticity. *J. Neurosci.* **20**, 8443–8451 (2000). 11069952 10.1523/JNEUROSCI.20-22-08443.2000 PMC6773171

[250] Calabresi, P., Picconi, B., Tozzi, A. & Di Filippo, M. Dopamine-mediated regulation of corticostriatal synaptic plasticity. *Trends Neurosci.* **30**, 211–219 (2007). 17367873 10.1016/j.tins.2007.03.001

[251] Kerr, J. N. & Wickens, J. R. Dopamine D-1/D-5 receptor activation is required for long-term potentiation in the rat neostriatum in vitro. *J. Neurophysiol.* **85**, 117–124 (2001). 11152712 10.1152/jn.2001.85.1.117

[252] Shen, W., Flajolet, M., Greengard, P. & Surmeier, D. J. Dichotomous dopaminergic control of striatal synaptic plasticity. *Science* **321**, 848–851 (2008). 18687967 10.1126/science.1160575 PMC2833421

[253] Hernandez-Lopez, S., Bargas, J., Surmeier, D. J., Reyes, A. & Galarraga, E. D1 receptor activation enhances evoked discharge in neostriatal medium spiny neurons by modulating an L-type Ca2+ conductance. *J. Neurosci.* **17**, 3334–3342 (1997). 9096166 10.1523/JNEUROSCI.17-09-03334.1997 PMC6573659

[254] Fieblinger, T. et al. Cell type-specific plasticity of striatal projection neurons in parkinsonism and L-DOPA-induced dyskinesia. *Nat. Commun.* **5**, 5316 (2014). 25360704 10.1038/ncomms6316 PMC4431763

[255] Schultz, W. Multiple dopamine functions at different time courses. *Annu Rev. Neurosci.* **30**, 259–288 (2007). 17600522 10.1146/annurev.neuro.28.061604.135722

[256] Berke, J. D. What does dopamine mean? *Nat. Neurosci.* **21**, 787–793 (2018). 29760524 10.1038/s41593-018-0152-y PMC6358212

[257] da Silva, J. A., Tecuapetla, F., Paixão, V. & Costa, R. M. Dopamine neuron activity before action initiation gates and invigorates future movements. *Nature* **554**, 244–248 (2018). 29420469 10.1038/nature25457

[258] Panigrahi, B. et al. Dopamine is required for the neural representation and control of movement vigor. *Cell* **162**, 1418–1430 (2015). 26359992 10.1016/j.cell.2015.08.014

[259] Kim, H. R. et al. A unified framework for dopamine signals across timescales. *Cell* **183**, 1600–1616.e1625 (2020). 33248024 10.1016/j.cell.2020.11.013 PMC7736562

[260] Bamford, N. S., Wightman, R. M. & Sulzer, D. Dopamine’s effects on corticostriatal synapses during reward-based behaviors. *Neuron* **97**, 494–510 (2018). 29420932 10.1016/j.neuron.2018.01.006 PMC5808590

[261] Prager, E. M. et al. Dopamine oppositely modulates state transitions in striosome and matrix direct pathway striatal spiny neurons. *Neuron* **108**, 1091–1102.e1095 (2020). 33080228 10.1016/j.neuron.2020.09.028 PMC7769890

[262] Hamid, A. A., Frank, M. J. & Moore, C. I. Wave-like dopamine dynamics as a mechanism for spatiotemporal credit assignment. *Cell* **184**, 2733–2749.e2716 (2021). 33861952 10.1016/j.cell.2021.03.046 PMC8122079

[263] Perez, S. et al. Striatum expresses region-specific plasticity consistent with distinct memory abilities. *Cell Rep.* **38**, 110521 (2022). 35294877 10.1016/j.celrep.2022.110521

[264] Jorgensen, S. H. et al. Behavioral encoding across timescales by region-specific dopamine dynamics. *Proc. Natl. Acad. Sci. USA* **120**, e2215230120 (2023). 36749722 10.1073/pnas.2215230120 PMC9963838

[265] Coddington, L. T. & Dudman, J. T. Learning from action: reconsidering movement signaling in midbrain dopamine neuron activity. *Neuron* **104**, 63–77 (2019). 31600516 10.1016/j.neuron.2019.08.036

[266] Long, C. & Masmanidis, S. C. The learning primacy hypothesis of dopamine: reconsidering dopamine’s dual functions. *Front. Cell. Neuroscie.* **19**, 10.3389/fncel.2025.1538500 (2025). 10.3389/fncel.2025.1538500 PMC12037477 40302787

[267] Ragsdale, C. W. Jr. & Graybiel, A. M. Fibers from the basolateral nucleus of the amygdala selectively innervate striosomes in the caudate nucleus of the cat. *J. Comp. Neurol.* **269**, 506–522 (1988). 2453535 10.1002/cne.902690404

[268] Yoshizawa, T. & Funahashi, M. Dopamine release in striatal striosome compartments in response to rewards and aversive outcomes during classical conditioning in mice. *Neurosci. Res.* **212**, 61–68 (2025). 39515479 10.1016/j.neures.2024.11.002

[269] Friedman, A. et al. Chronic stress alters striosome-circuit dynamics, leading to aberrant decision-making. *Cell* **171**, 1191–1205.e1128 (2017). 29149606 10.1016/j.cell.2017.10.017 PMC5734095

[270] Lee, I. B. et al. Persistent enhancement of basolateral amygdala-dorsomedial striatum synapses causes compulsive-like behaviors in mice. *Nat. Commun.* **15**, 219 (2024). 38191518 10.1038/s41467-023-44322-8 PMC10774417

[271] Chon, U., Vanselow, D. J., Cheng, K. C. & Kim, Y. Enhanced and unified anatomical labeling for a common mouse brain atlas. *Nat. Commun.* **10**, 5067 (2019). 31699990 10.1038/s41467-019-13057-w PMC6838086

[272] Kuan, L. et al. Neuroinformatics of the Allen Mouse Brain Connectivity Atlas. *Methods* **73**, 4–17 (2015). 25536338 10.1016/j.ymeth.2014.12.013

[273] Wang, Q. et al. The Allen Mouse Brain Common Coordinate Framework: a 3D reference atlas. *Cell* **181**, 936–953.e920 (2020). 32386544 10.1016/j.cell.2020.04.007 PMC8152789

[274] Hogeveen, J. et al. What does the frontopolar cortex contribute to goal-directed cognition and action? *J. Neurosci.* **42**, 8508–8513 (2022). 36351824 10.1523/JNEUROSCI.1143-22.2022 PMC9665930

[275] Mansouri, F. A., Koechlin, E., Rosa, M. G. P. & Buckley, M. J. Managing competing goals - a key role for the frontopolar cortex. *Nat. Rev. Neurosci.* **18**, 645–657 (2017). 28951610 10.1038/nrn.2017.111

[276] Ferrucci, L. et al. Social monitoring of actions in the macaque frontopolar cortex. *Prog. Neurobiol.* **218**, 102339 (2022). 35963359 10.1016/j.pneurobio.2022.102339

[277] Chau, B. K. H., Law, C. K., To, J. Y. L., Shum, D. H. K. & Mars, R. B. Complex functions of human lateral frontopolar cortex. *Brain* **148**, 3833–3843 (2025). 40795209 10.1093/brain/awaf289 PMC12588682

[278] Levy, R. The prefrontal cortex: from monkey to man. *Brain* **147**, 794–815 (2024). 37972282 10.1093/brain/awad389 PMC10907097

[279] Ferry, A. T., Ongur, D., An, X. & Price, J. L. Prefrontal cortical projections to the striatum in macaque monkeys: evidence for an organization related to prefrontal networks. *J. Comp. Neurol.* **425**, 447–470 (2000). 10972944 10.1002/1096-9861(20000925)425:3<447::aid-cne9>3.0.co;2-v

[280] Rudebeck, P. H. & Murray, E. A. The orbitofrontal oracle: cortical mechanisms for the prediction and evaluation of specific behavioral outcomes. *Neuron* **84**, 1143–1156 (2014). 25521376 10.1016/j.neuron.2014.10.049 PMC4271193

[281] Noonan, M. P., Chau, B. K. H., Rushworth, M. F. S. & Fellows, L. K. Contrasting effects of medial and lateral orbitofrontal cortex lesions on credit assignment and decision-making in humans. *J. Neurosci.* **37**, 7023–7035 (2017). 28630257 10.1523/JNEUROSCI.0692-17.2017 PMC6705719

[282] Carmichael, S. T. & Price, J. L. Architectonic subdivision of the orbital and medial prefrontal cortex in the macaque monkey. *J. Comp. Neurol.* **346**, 366–402 (1994). 7527805 10.1002/cne.903460305

[283] Noonan, M. P. et al. Separate value comparison and learning mechanisms in macaque medial and lateral orbitofrontal cortex. *Proc. Natl. Acad. Sci. USA* **107**, 20547–20552 (2010). 21059901 10.1073/pnas.1012246107 PMC2996698

[284] Strait, C. E., Blanchard, T. C. & Hayden, B. Y. Reward value comparison via mutual inhibition in ventromedial prefrontal cortex. *Neuron* **82**, 1357–1366 (2014). 24881835 10.1016/j.neuron.2014.04.032 PMC4086796

[285] Levy, D. J. & Glimcher, P. W. The root of all value: a neural common currency for choice. *Curr. Opin. Neurobiol.* **22**, 1027–1038 (2012). 22766486 10.1016/j.conb.2012.06.001 PMC4093837

[286] Woon, E. P., Sequeira, M. K., Barbee, B. R. & Gourley, S. L. Involvement of the rodent prelimbic and medial orbitofrontal cortices in goal-directed action: a brief review. *J. Neurosci. Res.* **98**, 1020–1030 (2020). 31820488 10.1002/jnr.24567 PMC7392403

[287] Vogt, B. A. & Paxinos, G. Cytoarchitecture of mouse and rat cingulate cortex with human homologies. *Brain Struct. Funct.* **219**, 185–192 (2014). 23229151 10.1007/s00429-012-0493-3

[288] van Heukelum, S. et al. Where is cingulate cortex? A cross-species view. *Trends Neurosci.* **43**, 285–299 (2020). 32353333 10.1016/j.tins.2020.03.007

[289] Corbit, L. H. & Balleine, B. W. The role of prelimbic cortex in instrumental conditioning. *Behav. Brain Res.* **146**, 145–157 (2003). 14643467 10.1016/j.bbr.2003.09.023

[290] Hart, G. & Balleine, B. W. Consolidation of goal-directed action depends on MAPK/ERK signaling in rodent prelimbic cortex. *J. Neurosci.* **36**, 11974–11986 (2016). 27881782 10.1523/JNEUROSCI.1772-16.2016 PMC6604924

[291] Rudebeck, P. H., Saunders, R. C., Lundgren, D. A. & Murray, E. A. Specialized representations of value in the orbital and ventrolateral prefrontal cortex: desirability versus availability of outcomes. *Neuron* **95**, 1208–1220.e1205 (2017). 28858621 10.1016/j.neuron.2017.07.042 PMC5600902

[292] Buckley, M. J. et al. Dissociable components of rule-guided behavior depend on distinct medial and prefrontal regions. *Science* **325**, 52–58 (2009). 19574382 10.1126/science.1172377

[293] Izquierdo, A., Suda, R. K. & Murray, E. A. Bilateral orbital prefrontal cortex lesions in rhesus monkeys disrupt choices guided by both reward value and reward contingency. *J. Neurosci.* **24**, 7540–7548 (2004). 15329401 10.1523/JNEUROSCI.1921-04.2004 PMC6729636

[294] Sias, A. C. et al. A bidirectional corticoamygdala circuit for the encoding and retrieval of detailed reward memories. *Elife* **10**, e68617 (2021). 10.7554/eLife.68617 PMC8266390 34142660

[295] Haber, S. N., Kunishio, K., Mizobuchi, M. & Lynd-Balta, E. The orbital and medial prefrontal circuit through the primate basal ganglia. *J. Neurosci.* **15**, 4851–4867 (1995). 7623116 10.1523/JNEUROSCI.15-07-04851.1995 PMC6577885

[296] Isomura, Y., Ito, Y., Akazawa, T., Nambu, A. & Takada, M. Neural coding of “attention for action” and “response selection” in primate anterior cingulate cortex. *J. Neurosci.* **23**, 8002–8012 (2003). 12954861 10.1523/JNEUROSCI.23-22-08002.2003 PMC6740492

[297] Zeeb, F. D., Baarendse, P. J., Vanderschuren, L. J. & Winstanley, C. A. Inactivation of the prelimbic or infralimbic cortex impairs decision-making in the rat gambling task. *Psychopharmacology* **232**, 4481–4491 (2015). 26387517 10.1007/s00213-015-4075-y

[298] Malagon-Vina, H., Ciocchi, S., Passecker, J., Dorffner, G. & Klausberger, T. Fluid network dynamics in the prefrontal cortex during multiple strategy switching. *Nat. Commun.* **9**, 309 (2018). 29358717 10.1038/s41467-017-02764-x PMC5778086

[299] DiNicola, L. M., Sun, W. & Buckner, R. L. Side-by-side regions in dorsolateral prefrontal cortex estimated within the individual respond differentially to domain-specific and domain-flexible processes. *J. Neurophysiol.* **130**, 1602–1615 (2023). 37937340 10.1152/jn.00277.2023 PMC11068361

[300] De Pisapia, N., Slomski, J. A. & Braver, T. S. Functional specializations in lateral prefrontal cortex associated with the integration and segregation of information in working memory. *Cereb. Cortex* **17**, 993–1006 (2007). 16769743 10.1093/cercor/bhl010

[301] MacDonald, A. W., Cohen, J. D. 3rd, Stenger, V. A. & Carter, C. S. Dissociating the role of the dorsolateral prefrontal and anterior cingulate cortex in cognitive control. *Science* **288**, 1835–1838 (2000). 10846167 10.1126/science.288.5472.1835

[302] Calzavara, R., Mailly, P. & Haber, S. N. Relationship between the corticostriatal terminals from areas 9 and 46, and those from area 8A, dorsal and rostral premotor cortex and area 24c: an anatomical substrate for cognition to action. *Eur. J. Neurosci.* **26**, 2005–2024 (2007). 17892479 10.1111/j.1460-9568.2007.05825.x PMC2121143

[303] Noonan, M. P., Kolling, N., Walton, M. E. & Rushworth, M. F. Re-evaluating the role of the orbitofrontal cortex in reward and reinforcement. *Eur. J. Neurosci.* **35**, 997–1010 (2012). 22487031 10.1111/j.1460-9568.2012.08023.x

[304] Walton, M. E., Behrens, T. E., Noonan, M. P. & Rushworth, M. F. Giving credit where credit is due: orbitofrontal cortex and valuation in an uncertain world. *Ann. N.Y. Acad. Sci.* **1239**, 14–24 (2011). 22145871 10.1111/j.1749-6632.2011.06257.x

[305] Walton, M. E., Behrens, T. E., Buckley, M. J., Rudebeck, P. H. & Rushworth, M. F. Separable learning systems in the macaque brain and the role of orbitofrontal cortex in contingent learning. *Neuron* **65**, 927–939 (2010). 20346766 10.1016/j.neuron.2010.02.027 PMC3566584

[306] Chau, B. K. et al. Contrasting roles for orbitofrontal cortex and amygdala in credit assignment and learning in macaques. *Neuron* **87**, 1106–1118 (2015). 26335649 10.1016/j.neuron.2015.08.018 PMC4562909

[307] Duan, L. Y. et al. Controlling one’s world: Identification of sub-regions of primate PFC underlying goal-directed behavior. *Neuron* **109**, 2485–2498.e2485 (2021). 34171290 10.1016/j.neuron.2021.06.003 PMC8346232

[308] Jackson, S. A., Horst, N. K., Pears, A., Robbins, T. W. & Roberts, A. C. Role of the Perigenual Anterior Cingulate and Orbitofrontal Cortex in Contingency Learning in the Marmoset. *Cereb. Cortex* **26**, 3273–3284 (2016). 27130662 10.1093/cercor/bhw067 PMC4898677

[309] Sarafyazd, M. & Jazayeri, M. Hierarchical reasoning by neural circuits in the frontal cortex. *Science* **364**, eaav8911 (2019). 10.1126/science.aav8911 31097640

[310] Khamassi, M. et al. Rat anterior cingulate neurons responsive to rule or strategy changes are modulated by the hippocampal theta rhythm and sharp-wave ripples. *Eur. J. Neurosci.* **60**, 5300–5327 (2024). 39161082 10.1111/ejn.16496

[311] Horwitz, B. et al. Activation of Broca’s area during the production of spoken and signed language: a combined cytoarchitectonic mapping and PET analysis. *Neuropsychologia* **41**, 1868–1876 (2003). 14572520 10.1016/s0028-3932(03)00125-8

[312] Cooper, D. L. Broca’s arrow: evolution, prediction, and language in the brain. *Anat. Rec. B N. Anat.* **289**, 9–24 (2006). 10.1002/ar.b.20088 16437554

[313] Ferrari, P. F., Gerbella, M., Coudé, G. & Rozzi, S. Two different mirror neuron networks: the sensorimotor (hand) and limbic (face) pathways. *Neuroscience* **358**, 300–315 (2017). 28687313 10.1016/j.neuroscience.2017.06.052 PMC6063080

[314] Gerbella, M., Borra, E., Mangiaracina, C., Rozzi, S. & Luppino, G. Corticostriate projections from areas of the “lateral grasping network”: evidence for multiple hand-related input channels. *Cereb. Cortex* **26**, 3096–3115 (2016). 26088968 10.1093/cercor/bhv135

[315] Hart, E. E., Blair, G. J., O’Dell, T. J., Blair, H. T. & Izquierdo, A. Chemogenetic modulation and single-photon calcium imaging in anterior cingulate cortex reveal a mechanism for effort-based decisions. *J. Neurosci.* **40**, 5628–5643 (2020). 32527984 10.1523/JNEUROSCI.2548-19.2020 PMC7363467

[316] Takeuchi, D. et al. Cingulate-motor circuits update rule representations for sequential choice decisions. *Nat. Commun.* **13**, 4545 (2022). 35927275 10.1038/s41467-022-32142-1 PMC9352796

[317] Lazari, A. et al. The mouse motor system contains multiple premotor areas and partially follows human organizational principles. *Cell Rep.* **43**, 114191 (2024). 38717901 10.1016/j.celrep.2024.114191 PMC7617973

[318] Sul, J. H., Jo, S., Lee, D. & Jung, M. W. Role of rodent secondary motor cortex in value-based action selection. *Nat. Neurosci.* **14**, 1202–1208 (2011). 21841777 10.1038/nn.2881 PMC3164897

[319] Takada, M., Tokuno, H., Nambu, A. & Inase, M. Corticostriatal projections from the somatic motor areas of the frontal cortex in the macaque monkey: segregation versus overlap of input zones from the primary motor cortex, the supplementary motor area, and the premotor cortex. *Exp. Brain Res.* **120**, 114–128 (1998). 9628410 10.1007/s002210050384

[320] Bakken, T. E. et al. Comparative cellular analysis of motor cortex in human, marmoset and mouse. *Nature* **598**, 111–119 (2021). 34616062 10.1038/s41586-021-03465-8 PMC8494640

[321] Kakei, S., Hoffman, D. S. & Strick, P. L. Muscle and movement representations in the primary motor cortex. *Science* **285**, 2136–2139 (1999). 10497133 10.1126/science.285.5436.2136

[322] Graziano, M. S., Taylor, C. S. & Moore, T. Complex movements evoked by microstimulation of precentral cortex. *Neuron* **34**, 841–851 (2002). 12062029 10.1016/s0896-6273(02)00698-0

[323] Georgopoulos, A. P., Kalaska, J. F., Caminiti, R. & Massey, J. T. On the relations between the direction of two-dimensional arm movements and cell discharge in primate motor cortex. *J. Neurosci.* **2**, 1527–1537 (1982). 7143039 10.1523/JNEUROSCI.02-11-01527.1982 PMC6564361

[324] Georgopoulos, A. P., Schwartz, A. B. & Kettner, R. E. Neuronal population coding of movement direction. *Science* **233**, 1416–1419 (1986). 3749885 10.1126/science.3749885

[325] Brown, V. J. & Tait, D. S. Attentional set-shifting across species. *Curr. Top. Behav. Neurosci.* **28**, 363–395 (2016). 26873018 10.1007/7854_2015_5002

[326] Tait, D. S., Bowman, E. M., Neuwirth, L. S. & Brown, V. J. Assessment of intradimensional/extradimensional attentional set-shifting in rats. *Neurosci. Biobehav Rev.* **89**, 72–84 (2018). 29474818 10.1016/j.neubiorev.2018.02.013

[327] Weed, M. R., Bryant, R. & Perry, S. Cognitive development in macaques: attentional set-shifting in juvenile and adult rhesus monkeys. *Neuroscience* **157**, 22–28 (2008). 18805462 10.1016/j.neuroscience.2008.08.047

[328] Weed, M. R. et al. Performance norms for a rhesus monkey neuropsychological testing battery: acquisition and long-term performance. *Brain Res. Cogn. Brain Res.* **8**, 185–201 (1999). 10556598 10.1016/s0926-6410(99)00020-8

[329] Kuwabara, M., Kang, N., Holy, T. E. & Padoa-Schioppa, C. Neural mechanisms of economic choices in mice. *Elife* **4**, e49669 (2020). 10.7554/eLife.49669 PMC7062473 32096761

[330] Barker, J. M., Glen, W. B., Linsenbardt, D. N., Lapish, C. C. & Chandler, L. J. Habitual Behavior Is Mediated by a Shift in Response-Outcome Encoding by Infralimbic Cortex. *eNeuro* **9**, 10.1523/eneuro.0337-17.2017 (2017). 10.1523/ENEURO.0337-17.2017 PMC5752702 29302616

[331] Shipman, M. L., Trask, S., Bouton, m. E. & Green, J. T. Inactivation of prelimbic and infralimbic cortex respectively affects minimally-trained and extensively-trained goal-directed actions. *Neurobiology of learning and memory* **155**, 164–172 (2018). 10.1016/j.nlm.2018.07.010 PMC6345614 30053577

